# Comparative Studies of Extracts Obtained from *Brassica oleracea* L. Plants at Different Stages of Growth by Isolation and Determination of Isothiocyanates: An Assessment of Chemopreventive Properties of Broccoli

**DOI:** 10.3390/molecules29020519

**Published:** 2024-01-20

**Authors:** Magdalena Ligor, Małgorzata Szultka-Młyńska, Katarzyna Rafińska, Agata Cwudzińska

**Affiliations:** Chair of Environmental Chemistry and Bioanalytics, Faculty of Chemistry, Nicolaus Copernicus University, 7 Gagarina Street, 87-100 Toruń, Poland; mszultka@chem.umk.pl (M.S.-M.); katraf@umk.pl (K.R.); agatacwudzinska@gmail.com (A.C.)

**Keywords:** isothiocyanates, sulforaphane, extraction, spectroscopy, liquid chromatography, mass spectrometry, chemopreventive

## Abstract

The main goal of this work was to develop analytical procedures for the isolation and determination of selected isothiocyanates. As an example, particularly sulforaphane from plants of the *Brassicaceae Burnett* or *Cruciferae* Juss family. The applied methodology was mainly based on classical extraction methods and high-performance liquid chromatography coupled with tandem mass spectrometry. Moreover, the effect of temperature on the release of isothiocyanates from plant cells was considered. The cytotoxic activity of the obtained plant extracts against a selected cancer cell line has also been included. The results allow evaluating the usefulness of obtained plant extracts and raw sprouts regarding their content of isothiocyanates—bioactive compounds with chemopreventive properties.

## 1. Introduction

**Figure 1:**
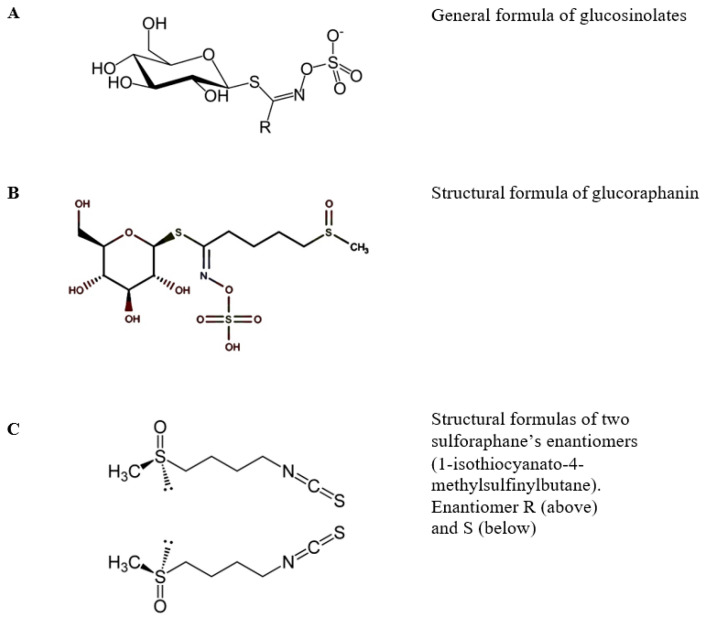
Structures of glucosinolates (**A**), glucoraphanin (**B**), and sulforaphane (**C**).

Plants from the cruciferous family (*Brassicaceae* syn. *Cruciferae*) are a rich source of active substances with chemopreventive and antioxidant properties [1,2,3,4]. A significant part of recent research has been focused on studying extracts obtained from those plants in order to assess their usefulness related to the content of isothiocyanates, which belong to the group of biologically active compounds [5,6]. A large volume of epidemiological data demonstrates that increasing the consumption of *Brassicaceae* vegetables can lower the risk of developing cancer of the lung [7], pancreas [8], colon [9], prostate [10], and bladder [11]. The available study results also suggest that including cruciferous plants in one’s diet can mitigate symptoms of diabetes, cardiovascular diseases, neurodegenerative diseases, and inflammation-related disorders [12,13]. The majority of published materials on the health benefits of a *Cruciferae*-rich diet point to sulforaphane (1-isothiocyanato-4-(methylsulfinyl)butane) as the most active phytochemical among isothiocyanates [14]. The precursors of active isothiocyanates are glucosinolates, a group of organic compounds that are a type of thioglycoside. The number of currently known substances classified in this group exceeds 120. They have a similar structure that includes a β-D-thioglucose molecule, a sulfone oxime group, and a side chain derived from one of seven amino acids (Figure 1) [15,16].

**Figure 2:**
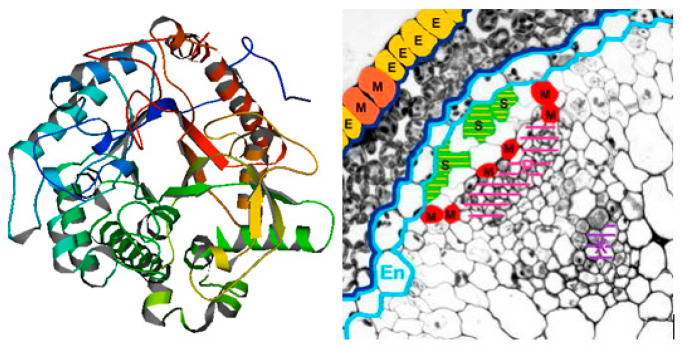
Structure of myrosinase, PDB code: 2 myr, E.C. no. 3.2.3.1., and its location in plant tissue, where M—myrosine cells; S—vacuoles containing glucosinolates; E—cells containing epithiospecifier protein ESP (protein data bank: https://www.rcsb.org/structure/removed/2myr (accessed on 12 December 2023); Kissen et al., 2009) [23].

Glucosinolates are secondary metabolites characteristic of plants from the cabbage (*Brassicaceae*), caper (*Capparaceae*), and papaya (*Caricaceae*) families. Glucosinolates can be found in various elements of plant structure: within the shoot system in leaves, stems, and seeds, and underground in roots and rhizomes [15,16]. Depending on the environment in which the plant is cultivated (location, soil fertility, temperature, irrigation level, and fertilization type), as well as the plant genotype, the glucosinolate content can vary significantly between individual species and cultivars [17]. The highest concentrations were found in the young sprouts of cruciferous vegetables. Tests in vitro and in vivo (on animals) suggest that isothiocyanates, which are products of the enzymolysis of glucosinolates, are beneficial to health. Considering the positive data obtained from epidemiological research, sulforaphane is the most extensively studied isothiocyanate compound [18]. Sulforaphane is created through the enzymatic hydrolysis of glucoraphanin, its biologically inactive precursor. The catalyst of this reaction is the endogenous plant enzyme myrosinase. Sulforaphane was first isolated and identified in 1959 from Savoy cabbage, red cabbage, and cress [19,20]. However, it did not attract much attention until 1992, when extracts of multiple plants were obtained and tested with regard to the induction of phase II enzymes, which have a significant role in carcinogenetic and mutagenic processes [21]. Sulforaphane naturally occurs as two optical isomers due to the presence of an asymmetric atom of sulfur in its structure (Figure 1). Naturally occurring sulforaphane is a yellow, oily substance soluble in water, acetonitrile, methanol, ethanol, ethyl acetate, dichloromethane, and chloroform. In aqueous solutions at increased temperature, it can degrade into volatile products such as dimethyl disulfide, S-methyl methylthiosulfinate, 1,2,4-trithiolane, 4-isothiocyanato-1-methylthio-1-butene, and 3-butenyl isothiocyanate [22]. Compared to other phytochemicals, this compound is a relatively small molecule (MW 177.29 g/mol), which, among others, makes sulforaphane highly bioavailable. Undamaged plant tissues do not contain free sulforaphane but glucoraphanin, its biologically inactive precursor. The process of converting inactive glucoraphanin into sulforaphane takes place when plant tissue is damaged due to chewing or cutting fresh plants. A breach of tissue integrity causes the release of the enzyme myrosinase (β-thioglucosidase), a glycoprotein in the tissues of cruciferous plants that can be found in myrosine cells (Figure 2). The location of those cells varies depending on the specific part of the plant structure and on its growth stage [23].

**Figure 3:**
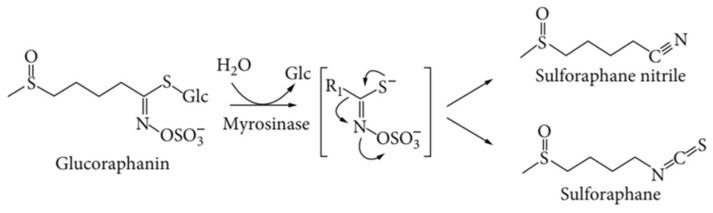
Scheme of reaction for glucoraphanin hydrolysis catalyzed by the enzyme myrosinase, which produces sulforaphane and its nitrile derivative [26].

Damage to plant tissue leads to the contact of myrosinase with glucosinolates present in vacuoles (Figure 3). This causes enzymatic hydrolysis of glucoraphanin thioglucoside bonds; this creates an unstable intermediate product (thiohydroxime-O-sulfonic aglucone), which—depending on the reaction environment and the presence of protein factors—transforms on its own into isothiocyanates, thiocyanates, nitriles, or epithionitriles. In the case of glucoraphanin, two main products are created: bioactive sulforaphane and its nitrile derivative (Figure 3) [22,24,25,26].

To fully evaluate the health benefits resulting from the consumption of cruciferous plants, we need to develop suitable methods for isolating, purifying, and determining isothiocyanates in plant material. The methodology of sample preparation for isolating isothiocyanates from plant material and further quantitative and qualitative analysis is a very important step where all factors, such as temperature, pH, and hydrolysis duration, have to be selected with great care. This will be explained in detail in the following study (see Results and Discussion). As this group of substances is the focus of considerable interest, different chromatographic methods have been developed in order to determine isothiocyanates both in plant extracts and in plant material [27,28]. So far, the techniques most frequently used to separate and identify isothiocyanates in plant extracts include gas chromatography in tandem with mass spectrometry (GC/MS) [6,29], liquid chromatography with a UV spectrophotometer (HPLC/UV) [30,31], and liquid chromatography in tandem with mass spectrometry (HPLC/MS) [5]. Also, a sensitive, relatively simple, and low-cost method that uses 1,2-benzenedithiol as a reagent has been developed for the quantitative determination of isothiocyanates as well as their metabolites [32].

The objective of the present study was to develop methodologies for isolating and determining selected isothiocyanates from *Brassica oleracea* L. plants as well as to assess the cytotoxic activity of the obtained plant extracts against a selected cancer cell line. Thus, various extraction methods are employed to achieve the best possible yield of bioactive components from plant material. Maceration is a classic extraction method that has been widely used for the isolation of isothiocyanates due to their availability, efficiency, and comprehensive applicability. The conventional extraction techniques using methanol and ethanol and supercritical fluid extraction (SFE) have been compared. In this study, we developed a quick and robust method to determine sulforaphane. The results allow for evaluating the usefulness of the obtained plant extracts and raw sprouts with regard to their content of isothiocyanates—bioactive compounds with chemopreventive properties.

## 2. Results and Discussion

### 2.1. The Isolation and Determination of Isothiocynates from Plant Material

An important step in the research was the development of appropriate procedures that would allow the characterization of the glucosinolate hydrolysis products. Hence, the goal of creating the methodology for isolating and determining isothiocyanates in plant material is to intentionally increase glucosinolate conversion to the desired final product of the reaction [33]. Proper preparation of raw plants’ material, which contains various groups of compounds for analysis, is an important and complex process with preliminary steps such as selection, harvesting, drying, lyophilization, and grounding (homogenization) of the studied plant material [34]. Intending to develop the methodology for determining isothiocyanates in plant material, the authors took into consideration the fact that these substances do not occur in undamaged plant tissue. Thus, a necessary step preceding the extraction of analytes from the samples was to cut the plant material into small pieces in order to release myrosinase, the enzyme that is a catalyst for the hydrolysis of glucosinolates to isothiocyanates. The hydrolysis was carried out in an appropriate environment which allowed increasing the number of bioactive products while simultaneously minimalizing the conversion of glucosinolates to compounds without health benefits. Therefore, sample preparation for isolating biologically active substances and further quantitative and qualitative analysis of isothiocyanates was a very important step. All factors, such as temperature, pH, and hydrolysis duration, had to be selected with great care. The results obtained in other laboratories were of great help [35]. Considering the hydrolysis reaction to be the crucial step during quantitative determination of sulforaphane in plant material, Campas-Baypoli and co-workers attempted to optimize its conditions with the use of response surface methodology (RSM) [30]. The variables used in their studies were such parameters as pH (3, 4, 5, 6), proportion of sample volume to acid water used (1:2, 1:4, 1:6), and hydrolysis duration (2, 4, 6 h). The temperature was consistently kept at 45 °C. The results of statistical analysis demonstrated that the factor that had the greatest impact on the efficiency of sulforaphane extraction (in addition to the selection of a suitable solvent) was the hydrolysis pH 6; such a hydrolysis reaction environment was therefore used in the quantitative determination of sulforaphane. Considering the above, our study tested the influence of temperature on the degree of glucoraphanin conversion to sulforaphane. The plant material in this experiment were six-day-old broccoli sprouts, processed in one of the three ways: (1) lyophilization of raw sprouts; (2) gentle thermal processing at 60 °C for 10 min and then lyophilization; (3) processing with steam (temp. 100 °C, 5 min), followed by lyophilization. Thus, the obtained lyophilizates were hydrolyzed and extracted with dichloromethane. Quantitative analysis was performed with HPLC-DAD, and on this basis, the degree of glucoraphanin conversion to sulforaphane was determined. Detailed results will be presented in one of the following subsections.

The obtained extracts needed to be further purified and enriched in order to eliminate undesirable substances (interferents) from the starting extract, as such substances could negatively affect separation and detection during the subsequent chromatographic analysis. Thus, a number of studies turned to solid phase extraction (SPE), used for isolating and concentrating analytes from the liquid sample as well as for moving them to the solid phase and ensuring they stay there [34]. The broad selection of sorbents used as solid phases in this method allows precise and selective isolation of substances based on various physicochemical interactions between the analyte and the solid phase. The most popular materials used in SPE extraction include silica and polymer sorbents modified with different groups [36]. Solid phase extraction was successfully used to isolate sulforaphane from broccoli. An attempt was made to optimize the SPE conditions [31] by using three types of sorbents: silica, silica modified with octadecyl groups (C18), and silica modified with amino groups. The solvents used for elution included water, acetonitrile, dichloromethane, ethyl acetate, hexane, and acetic acid. More sulforaphane was extracted with silica sorbent than with the other two solid phases due to the weakly polar character of the isolated isothiocyanate, which is selectively adsorbed on a sorbent with similar polarity [31]. Furthermore, the ethyl acetate used as a solvent for washing the sample did not elute sulforaphane, demonstrating that this is a suitable solution for washing interferents out of the sample. The best eluting solvent turned out to be dichloromethane, which allows quantitative isolation of sulforaphane from the sample. When selecting an appropriate technique, researchers should consider the chemical structure of the analyte, as this determines the choice of the most selective solvent. Extraction methods should also be efficient, relatively simple, cost-effective, and environmentally friendly [37]. Therefore, we decide that bioactive substances from plant materials can be isolated with classical extraction techniques, such as commonly used maceration. The efficiency of this type of extraction depends mainly on the solvent used, where the most important selection criteria are its polarity and capacity to solve the analyte. However, like other classical extraction techniques, maceration has two main drawbacks: low selectivity and the need for large amounts of toxic solvent. Hence, today we can observe the development of extraction techniques based on non-toxic or low-toxic solvents, such as supercritical fluid extraction (SFE). The highest efficiency of extraction was achieved for maceration with methanol (within the range of 11 to 36%). Slightly lower values were obtained for extraction using ethanol (from 5.6 to 14%), and the least effective was SFE extraction with 96% ethanol added as co-solvent (from 2.3 to 4.4%). The effectiveness of the extraction process depends predominantly on matching the solvent to the chemical characteristics of the extracted substances. Plant material is a rich, complex matrix containing compounds of different structures and physicochemical properties. Many secondary plant metabolites, which are often biologically active, are polar. The best results achieved during extraction with methanol can be attributed to the fact that the matrix of the studied plant material obtained from sprouts and mature broccoli is rich in polar compounds. At this stage, extraction was not selective with regard to isothiocyanates but made it possible to isolate a number of other polar substances. Yet the best extraction technique is not always the most effective one if many interferents are retained as well. Hence, an important step preceding the analysis of selected compounds is the procedure of purifying and enriching the obtained extracts with SPE. Due to the high extraction efficiency of methanol, we decided to use methanolic extracts in subsequent studies.

### 2.2. Determining the Total Content of Isothiocyanates in Plant Extracts—Cyclocondensation with 1,2-Benzenedithiol

The analytical method based on the reaction of isothiocyanate cyclocondensation was The analytical method based on the reaction of isothiocyanate cyclocondensation was developed by Zhang, working on cancer chemoprevention at Roswell Park Comprehensive Cancer Center [32]. The method uses the ability of isothiocyanates to react quantitatively with 1,2-benzenedithiol. The product of this reaction is cyclic thiocarbonyl and free amines. The mechanism of cyclocondensation uses the fact that the carbon in the –NCS group of isothiocyanates is strongly electrophilic and is attacked first by one and then the other thiol group in the structure of dithiol.

Quantitative determination of isothiocyanates via cyclocondensation uses the ability of the produced 1,3-benzenedithiole-2-thione to absorb radiation in the UV range. The maximum absorbance was determined for the wavelength of 365 nm. The product is created quickly and is stable at different temperatures, while the reaction itself is very sensitive, relatively simple, and inexpensive. In this way, we can determine both isothiocyanates and their metabolites, and the method can be used to study the bioavailability of those compounds [32].

The method applied in our study, starting from cyclocondensation with 1,2-benzenedithiol used as solvent and ending with spectrophotometric analysis of the obtained samples, made it possible to determine the general content of isothiocyanates in the investigated plant extracts, which included methanolic extracts made from broccoli sprouts (lab-grown and shop-purchased) and from a mature broccoli head. The plant material was also subjected to thermal processing (at 60 °C and 100 °C) in order to evaluate the impact of increased temperature on isothiocyanate content in plants. In addition to methanol extracts, the authors prepared extracts made with supercritical solvent as well as ethanol extracts for three selected samples. In order to compare the content of isothiocyanates in different cruciferous vegetables, the analysis also included kale sprouts and radish sprouts dried at 30 °C. Samples of broccoli sprouts dried at 30 °C were prepared as well in order to assess the impact of the drying process on the isothiocyanate content of the plant material.

Sulforaphane was used as a standard isothiocyanate, and its solutions were used to obtain the calibration curve (supplementary file, Figure S1). It illustrates the relation between absorbance and concentration in the sample. The absorbance values determined for standard solutions were reduced by the absorbance value of the control sample that did not contain sulforaphane solution.

Based on the regression curve equation, which is characterized by linearity for the concentration range of 4–80 µg/mL, the concentration of isothiocyanates in the tested plant samples was calculated as sulforaphane equivalent. The absorbance of the tested samples was decreased by the results of the blind test made for all solutions without 1,2-benzenedithiol. The summary of absorbance values measured for individual samples as well as the calculated isothiocyanate concentrations (µg/mL) are presented in Table S1 (supplementary file). It turned out that steam processing decreased conversion to 4%. In the sprouts that were not subjected to any processing, the level of glucosinolate conversion was 23%, while the highest degree of conversion—98%—was determined for sprouts heated to 60 °C. The quantitative results based on the weight of the raw plant material are presented later in this work.

### 2.3. Chromatographic Techniques in Determining Isothiocyanates, Including Sulforaphane

As the chemopreventive potential of isothiocyanates (and of sulforaphane in particular) is a subject of great interest to researchers, various chromatographic methods have been developed to determine these substances both in plant extracts and biological material [31]. The techniques most frequently used to separate and identify isothiocyanates in plant extracts include gas chromatography in tandem with mass spectrometry (GC/MS) as well as liquid chromatography with a UV spectrophotometric detector (HPLC/UV) or mass spectrometry (HPLC/MS). However, the instability of sulforaphane during GC analysis due to the high temperature at the injection ports turned out to be a significant obstacle, so the use of those techniques in the quantitative determination of isothiocyanates has been gradually discontinued.

To identify and confirm the presence of sulforaphane and its metabolites in the analyzed extracts, the authors conducted a LC/MS analysis with electrospray ionization in positive and negative modes ((ESI+) (ESI−)).

The obtained mass spectra of the standard sulforaphane solution demonstrated characteristic signals coming from the pseudo-molecular sulforaphane ion and its metabolites. Due to the absence of relevant standard solutions, sulforaphane metabolites were determined in the tested samples. Figures S2 and S3 (supplementary file) summarize the fragmentation spectra obtained for the standard solution of sulforaphane and for its metabolites.

Based on the fragmentation spectra, Table 1 presents a summary of the *m/z* values of pseudo-molecular ions and product ions for individual compounds determined with the LC/MS technique.

### 2.4. Analysis of Plant Extracts

The content of sulforaphane and its metabolites in the tested samples was confirmed with the use of single-ion monitoring (SIM) mode. In this mode, signals corresponding to individual desirable ions are visible in the obtained spectrum. The mass spectra of all the tested extracts contained signals coming from sulforaphane and its metabolites. The glucoraphanin signal was not recorded only in the case of extract from raw broccoli sprouts purchased in a shop (E2). Figure S10 (supplementary file) presents example mass spectra with characteristic signals obtained for the extract of raw lab-grown broccoli sprouts (E1). Table 2 below contains a comparison of signals in SIM mass spectra for specific tested samples.

To observe the relations between the changing content of sulforaphane and glucoraphanin in the investigated extracts, the table below shows selected intensity values for signals coming from these compounds (Table 3).

The above summary reveals a relationship between the growing intensity of the signal coming from sulforaphane and the simultaneous decrease in intensity of the signal corresponding to glucoraphanin. In the case of sample E2 (shop-purchased sprouts, raw), no glucoraphanin signal was detected, so we may assume that it has been entirely converted into sulforaphane. Sulforaphane signals in the MS spectra have the highest intensity for extracts no. 7 and 8 (lab-grown and shop-purchased sprouts, respectively, 100 °C, 5 min). At the same time, the intensity of glucoraphanin signals remains low for these extracts, which confirms the relationship described above. LC/MS analysis additionally confirmed the results obtained with HPLC/UV. In the extracts for which the determined sulforaphane content was the highest (E2, E6, E7, and E8), MS analysis demonstrated that glucoraphanin was converted to a large degree or even entirely (E2) to sulforaphane. Meanwhile, for the extracts where sulforaphane content could not be determined or was low (E3, E4, E5, and E9), the LC/MS analysis in SIM mode and the intensities of signals obtained in this way demonstrated that the level of conversion of glucoraphanin to sulforaphane was low.

### 2.5. Validation of the Method for Sulforaphane Determination with HPLC/UV

Appropriate conditions for HPLC/UV were selected for the standard solution of sulforaphane with the lowest intensity (4 µg/mL). The mobile phase used was a mixture of two solvents, acetonitrile/water, at two volume ratios (60:40, 70:30). Better separation of peaks and shorter retention time were achieved for the acetonitrile/water (70:30 *v*/*v*) mobile phase. The validation also included verification of the impact of the UV detector wavelength on the sulforaphane peak in the chromatogram. For this purpose, two wave lengths were tested: λ = 254 and 205 nm, and the latter turned out to be more selective with regard to sulforaphane. A calibration curve was constructed based on the relation of the peak area to the standard solution concentration (µg/mL). To determine other validation parameters, the standard solution with the lowest concentration (4 µg/mL) was applied to the column, and the analysis was repeated five times for this concentration. Table 4 below presents the specific validation parameters.

### 2.6. Determination of Sulforaphane Content in the Tested Plant Samples

Prepared, purified, and concentrated plant extracts were subjected to HPLC/UV analysis in the conditions selected during the method validation stage. The peaks obtained in chromatograms were correlated with peaks coming from standard solutions. Based on the overlapping retention times, the presence of sulforaphane in the prepared plant extracts was qualitatively confirmed.

From peak areas and the regression equation of the calibration curve, sulforaphane concentrations (µg/g) were calculated for specific samples. The results are summarized in the table below (Table 5).

The highest concentrations of sulforaphane were determined for samples subjected to thermal processing at 100 °C for 5 min (E7–E9). The concentrations of sulforaphane in samples treated with a temperature of 60 °C (E4, E5) were below the LOD in the case of sprouts, but for mature broccoli, they were determined to be 57 µg/g. The content of the analyzed isothiocyanate varied for raw plant extracts, and the highest concentration was found in shop-purchased sprouts (E2, c = 121 µg/g). Raw lab-grown sprouts (E1) had a low content of sulforaphane, while in raw mature broccoli (E3), this substance was not detected at the level allowing precise determination. It can be stated without a doubt that sprouts contain higher concentrations of isothiocyanates than mature vegetables.

The above results lead to the conclusion that raising the processing temperature to 100 °C results in increased conversion of glucosinolates to isothiocyanates, due to which their content in the samples is higher. However, this conclusion suggests that the enzyme participating in the hydrolysis of these compounds was not deactivated during heating. This may result from too short a time (5 min) of thermal processing of plant material, which leads to the supposition that myrosinase retained its catalytic activity. Another important step during the preparation of samples for analysis was the hydrolysis reaction carried out in a specific reaction environment (pH = 6) for a specific time (2.5 h) and at a specific temperature (45 °C). Perhaps the conditions at this stage, which involved thermal processing, negatively influenced the sulforaphane content in raw samples.

The next important step was the purification and enrichment of samples with SPE. Preparing SPE extraction involves the selection of many parameters that can significantly impact the end results of chromatographic analysis. Such parameters include column packing, solvents for washing and elution, and the volume of these solvents. The high affinity of the eluted analyte for the sorbent can also lead to difficult and incomplete desorption.

The quantitative analysis carried out with HPLC/UV and the validation of this analytical method conducted by the authors allow them to conclude that this method is sensitive, selective with regard to sulforaphane, precise (RSD = 0.057), and repeatable (CV = 5.7%). The applied analytical technique shows linearity for concentrations in the range of 4–80 µg/mL as well as a high coefficient of determination R^2^, amounting to 0.9989. There is no doubt that quantitative analysis of isothiocyanate content in plant extracts with the HPLC/UV technique needs to be perfected further, considering the factors that may negatively impact the precision and repeatability of determining sulforaphane in plant extracts during sample preparation.

### 2.7. Evaluation of Antioxidant Activity of the Tested Plant Extracts

The antioxidant activity of individual extracts was determined with the commonly used DPPH^•^ method of capturing free radicals. The measured values of absorbance were used to calculate the antioxidant activity, RSA (radical scavenging activity), a parameter of an ability to sweep free radicals calculated as the difference in the absorbance determined for the solution of DPPH^•^ synthetic free radical, and the absorbance of the tested extract after reaction with DPPH^•^ divided by the absorbance of the DPPH^•^ solution (more details in the Section 3). The results of absorbance measurements for the prepared samples and the calculated RSA, together with the standard deviation, are summarized in the table below. The difference in absorbance values as a function of antioxidant concentration in a tested solution is usually linear, so the results obtained for real-life samples can be recalculated as Trolox (6-hydroxy-2,5,7,8-tetramethylchroman-2-carboxylic acid) equivalent. To calculate TEAC (Trolox equivalent antioxidant capacity), data obtained for standard solutions were used (Trolox concentrations ranged from od 0.1 to 0.8 mM). The concentration of antioxidants in the tested extracts was determined with the use of the equation of the calibration curve constructed for Trolox: y = −0.4655x + 0.5012, coefficient of determination R^2^ = 0.9942. Table 6 summarizes the results of the antioxidant concentrations in the samples expressed as Trolox equivalents. The highest concentration of antioxidant-type substances was determined for the samples containing plant raw materials dried at 30 °C. The most antioxidants were found in the extract from dried kale sprouts (90% RSA), while the content of these substances was only slightly lower in the extracts from lab-grown and shop-purchased broccoli sprouts and from radish sprouts (89%, 89%, and 87% RSA, respectively). Comparing the RSA% data for broccoli sprouts, both lab-grown and store-bought, and for mature broccoli, we may conclude that sprouts are richer in substances with antioxidant properties.

### 2.8. Bioavailability

The bioavailability of sulforaphane depends to a large degree on the enzymatic activity of myrosinase. Thermal processing of plants/vegetables—such as cooking, steaming, or microwaving—causes deactivation of the enzyme and decreases the amount of sulforaphane several times. It was demonstrated, however, that gentle heating (60–70 °C) has a positive impact on the content of isothiocyanates in vegetables due to selective inactivation of the ESP protein and the lack of influence of such temperatures on myrosinase activity. As a result, the inhibition of the activity of the ESP protein leads to decreased production of nitrile compounds with a simultaneous increase in the amount of isothiocyanates created. This is important for consumers who want to maximize the amount of ingested isothiocyanates for health benefits [14]. However, despite the deactivation of myrosinase in plant tissue due to overly high temperatures, the conversion of glucoraphanin to sulforaphane is still possible in mammals’ organisms. It has been proven that the source of isothiocyanates in human organisms is the microbiological breakdown of glucosinolates by gut bacteria, more specifically, by the activity of thioglucosidase in the intestinal flora. Still, it has been demonstrated that the bioavailability of SFN is six times lower when glucosinolate hydrolysis does not occur before the plants are consumed [16]. The microbiological breakdown of glucosinolates also differs between individuals due to the variability of intestinal flora. Thus, therapy with antibiotics or mechanical cleansing of the intestines may limit the bioavailability of isothiocyanates [15,24].

### 2.9. Biological Activity: Methods of Determining Cytotoxic Activity In Vitro

**Figure 4:**
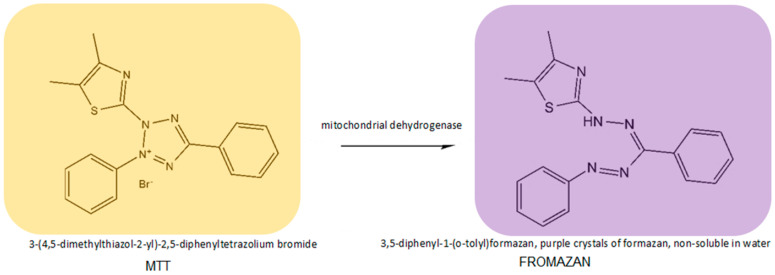
Reaction of reduction of MTT tetrazolium salt to formazan, catalyzed by mitochondrial dehydrogenase.

The biological activity of substances with chemotherapeutic potential is assessed through research on cell models in vitro (cytotoxic activity) or on animals in vivo (anti-cancer activity). The advantages of using cell lines in cytotoxicity testing include such factors as speed, ease of controlling cellular processes, repeatability, the opportunity to reduce the amounts of substances used, and the possibility of working on human cells. To determine the cytotoxic activity of a compound, a number of tests are conducted that allow measuring and controlling changes related to disruptions to physiological processes in cells that are caused by the investigated substance [38]. The type of test notwithstanding, the initial procedure is the same and involves incubating selected cell lines with the tested substance for a specific time at increasing concentrations. The next stage differs for individual tests due to the number of determined parameters, i.e., number of cells, cell division capability, mitochondrial activity, penetration of dyes into lysosomes, determination of total protein and DNA content in a cell, and DNA synthesis inhibition. The test most commonly used to determine the cytotoxicity of a given substance is the MTT assay [38,39]. The method is based on the capability of the enzyme (mitochondrial dehydrogenase) to convert orange, water-soluble tetrazolium salt (3-(4,5-dimethylthiazol-2-yl)-2,5-diphenyltetrazolium bromide) into a reduced form—purple crystals of formazan, non-soluble in water (Figure 4).

This type of reduction reaction, with the participation of mitochondrial dehydrogenase, occurs only in living cells. After dissolving formazan crystals, the enzymatic activity is determined by measuring the absorbance of the resulting solution. The amount of colorful, reduced MTT is proportional to the oxidizing activity of the cell’s mitochondria, which in turn determines the number of metabolically active (living) cells. The measure of the cytotoxic activity of a given compound is the determination of the inhibitory concentration, IC50, at which the growth and proliferation of cells in a culture are inhibited by 50% with regard to the growth of the control cells. To determine this value, a graph should be made to illustrate the relation of the tested parameter to the concentration of the tested substance.

In order to test the relationship between the content of isothiocyanates in the tested extracts obtained from cruciferous plants and their cytotoxic activity, a colorimetric MTT assay was performed. The anti-cancer activity of the tested extracts was assessed for the human colon adenocarcinoma cell line Caco-2. To evaluate safety and exclude the possibility of resulting undesirable activities, the cytotoxicity test was also performed for the mouse fibroblast cell line L929, approved by an ISO norm (EN ISO 10993-5). The cell line L929 is routinely used for testing the cytotoxic properties of medical products. Data on the viability of the L929 and Caco-2 series are combined in Table 7.

The data included in the table above lead to the conclusion that the extract obtained from mature, raw broccoli at a concentration of 5 mg/mL had the most visible cytotoxic effect on Caco-2 cells (cell viability 45%). Inhibited proliferation of colon adenocarcinoma cells (by ca. 40%) was also observed for extracts obtained from raw broccoli sprouts and mature broccoli after thermal processing at 60 and 100 °C (c = 5 mg/mL). The lowest cytotoxic ability was demonstrated by the extract of broccoli sprouts heated to 100 °C (cell viability 99%). This can result from the fact that during thermal processing, the enzyme converting glucosinolates to isothiocyanates, which can potentially show chemopreventive activity, was deactivated. It seems there is a positive relation between the amount of isothiocyanates in extracts and cytotoxic activity against Caco-2 cells in the case of extracts obtained from raw mature broccoli, raw broccoli sprouts, and mature broccoli subjected to thermal processing at 60 and 100 °C (c = 5 mg/mL).

**Figure 5:**
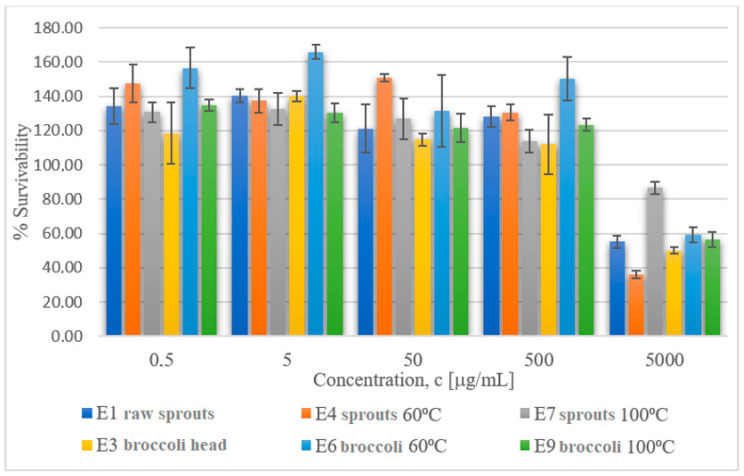
Cytotoxic activity of extracts obtained from broccoli sprouts and from mature broccoli towards the cell line L929.

**Figure 6:**
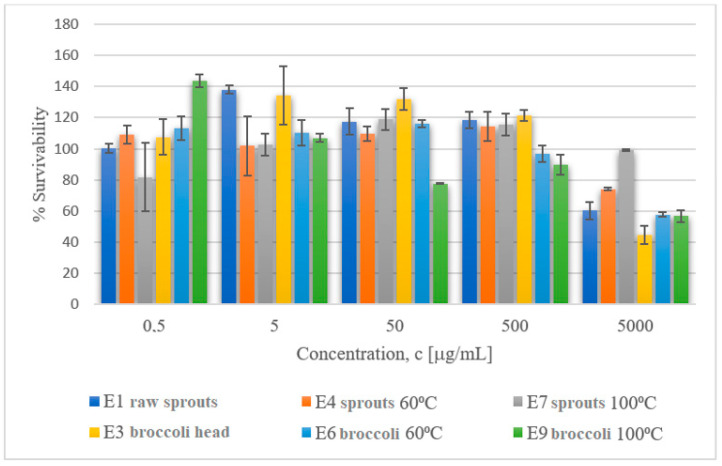
Cytotoxic activity of extracts obtained from broccoli sprouts and from mature broccoli towards the cell line Caco-2.

The data included in Table 7 on the viability of mouse fibroblast cell line L929 after exposition to extracts obtained from cruciferous plants demonstrate that the extract concentration of 5 mg/mL resulted in cell viability decreasing to ca. 60% for extracts obtained from raw broccoli sprouts and mature broccoli subjected to thermal processing at 60 and 100 °C (c = 5 mg/mL) and to ca. 50% for extracts from raw broccoli sprouts and raw mature broccoli. The lowest impact on L929 viability was made by the extract of broccoli sprouts subjected to thermal processing at 100 °C. Studies by Kołodziejski et al. (2022) with pure GL or ITC on human colon adenocarcinoma (HT29) cells showed that pure ITC/indole solutions showed the strongest antiproliferative activity. In our study, cells were treated with extracts consisting of a number of bioactive compounds that could modify the biological activity of the tested compounds [40]. Figure 5 and Figure 6 below present in graphic form the comparison of the viability of L929 and Caco-2 cells after exposure to the tested extracts obtained from cruciferous plants.

**Figure 7:**
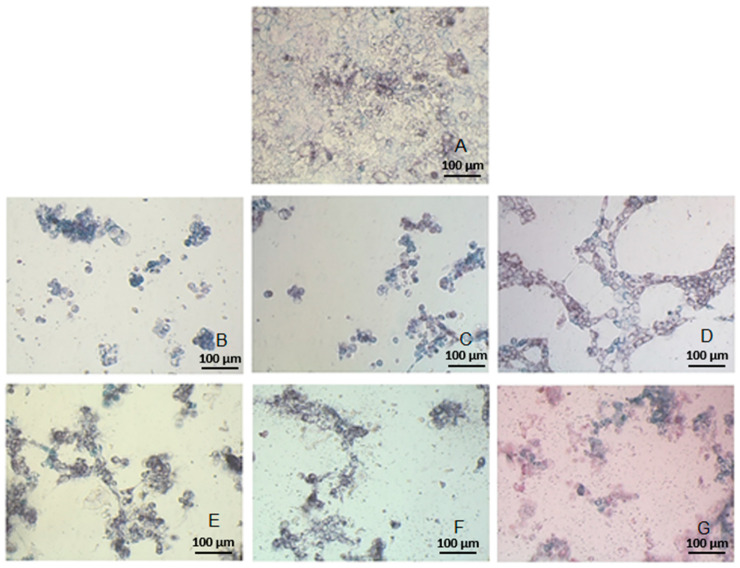
Caco-2 cells after 48 h exposure to extracts obtained from: (**B**) (E1, raw broccoli sprouts), (**C**) (E4, broccoli sprouts at 60 °C), (**D**) (E7, broccoli sprouts at 100 °C), (**E**) (E3, raw mature broccoli), (**F**) (E6, mature broccoli at 60 °C), (**G**) (E9, mature broccoli at 100 °C), and control: (**A**).

To illustrate the viability of Caco-2 cells, photographs were taken with the Olympus CKX41, an optical microscope with phase contrast, after treatment with MTT (Figure 7). Figure 7A–G present the photographs of the investigated cancer cells after 48 h exposure to specific extracts at a concentration of 5 mg/mL, where: A—control sample containing fresh growth medium instead of plant extract; B—extract E1, obtained from raw, lab-grown broccoli sprouts; C—extract E4 from lab-grown broccoli sprouts processed at 60 °C; D—extract E7 from lab-grown broccoli sprouts processed at 100 °C; E—extract E3 from raw mature broccoli; F—extract E6 from mature broccoli processed at 60 °C; G—extract E9 from mature broccoli processed at 100 °C.

In photographs B-G, the number of Caco-2 cancer cells is visibly lower than in the control sample, which did not contain any of the tested extracts. Cell clusters with a decreased number of cells were observed in the entire well of a multi-well PCR plate. The photographs taken with a microscope enabled visualization of the spectrophotometric results of toxicity measurements; they additionally confirmed a positive relationship between the isothiocyanate content of extracts and their cytotoxic activity against Caco-2 cells.

Depending on the carcinogenesis stage, sulforaphane affects many molecular mechanisms. Prevention of tumor initiation can be achieved by inhibiting the activation of carcinogenic factors or by their detoxification and removal from the organism. Blocking this stage of oncogenesis occurs through modulation of the activity of phase I enzymes that take part in transforming pro-carcinogens into carcinogens and through induction of phase II enzymes, responsible for detoxification of such oncogenic xenobiotics and their removal from the organism. When tumor growth has already been initiated, sulforaphane can act by disrupting the paths of various signals controlling cell proliferation, differentiation, and apoptosis and through regulation of the level of oxidative stress [25,41].

### 2.10. Modulation of the Activity of Phase I and II Enzymes

It has been demonstrated that products containing glucoraphanin or sulforaphane accelerate the process of detoxification and excretion of potential carcinogens from the body. Sulforaphane inhibits the activity of various isoforms of the CYP enzyme (CYP1A1, CYP2B1, and CYP3A4), which was observed during research on rat and human hepatocytes [25]. In turn, decreasing the level of transcripts of these enzymes within a cell results in decreased reactivity of pro-carcinogens. During phase II of metabolizing xenobiotics, sulforaphane induces such enzymes as heme oxygenase, quinone dehydrogenase, glutathione reductase, and glutathione peroxidase. These enzymes are catalysts in reactions that combine intermediate metabolites of carcinogens with body metabolites, which facilitates the removal of the former from the organism [22].

### 2.11. Stopping the Cell Cycle

Excessive proliferation is a characteristic of cancer cells. It is caused in cancerous cells by disruption of the activity of so-called control points, which are responsible for the course of specific phases of the cell cycle. The majority of cancer cells have an inactive control point in phase G1 as well as in phases G2 and M [42]. The control point of phase G1/S prevents replication of damaged DNA, while during phase G2/M, damaged chromosomes are selected so that they do not get into cells during mitosis. The role of chemopreventive factors involves inhibiting the processes of growth and proliferation of cancer cells by modulating the expression and/or activation of regulatory proteins of the cell cycle, such as cyclin D1, cyclin B1, cyclin-dependent kinase (CDK), as well as proteins p53, p21, and p27. Studies on several cell lines point to sulforaphane playing an important role in stopping the cell cycle, mostly in phase G2/M [43], but also in phase G1 [44] and S [45]. The cell-cycle regulating activity of sulforaphane is based, i.e., on inhibiting cyclin-dependent kinase (CDK), which, by creating a CDK/cyclin complex, facilitates the progression of the cell cycle. Another mechanism is the induction of protein p21, which acts as an inhibitor of the cell cycle [25]. Studies on colon cancer cell lines (HT-29 and Caco-2) demonstrated strong induction of protein p21 by sulforaphane in both cases [46].

### 2.12. Apoptosis

Apoptosis, or programmed cell death, plays a crucial role in maintaining tissue homeostasis and the elimination of damaged cells from the organism. Chemopreventive substances can influence many regulatory elements of this system, i.e., caspases, anti-apoptotic proteins of Bcl-2 family, and various transcription factors [46]. One of the most important components determining the course of apoptosis are enzymes called caspases, which can degrade genetic material by cutting peptide bonds. Research on human prostate cancer cells (DU145) demonstrated that exposition to sulforaphane for 24 h or longer significantly decreased the number of viable cells due to stopping phase G2/M and apoptotic death of cells, the proof of which was the increased release of DNA fragments connected to histones in prostate cancer cells [47]. A study using human colon cancer cells HCT116 demonstrated that administering 15 µM of sulforaphane resulted in activation of caspases 7 and 9 as well as influencing the expression of proteins from the Bcl-2 family [48].

### 2.13. Inhibiting Histone Deacetylase (HDAC)

What is becoming a promising area in cancer chemoprevention is epigenetic regulation of the expression of genes whose disruption leads to the growth of cancer. In addition to modification mechanisms, a significant role in epigenetic regulation of genes is ascribed to the reaction of histone acetylation/deacetylation, catalyzed by enzymes known as histone acetyltransferases (HAT) and histone deacetylases (HDAC) [49]. Increased activity and expression of HDAC have been observed in many cancer types; this leads to a decreased level of histone acetylation and, thus, the unnatural silencing of many genes during transcription [25].

Substances acting as histone deacetylase inhibitors (HDI), which induce increased acetylation of histones, are analyzed as a new generation of anti-tumor medicines. Increasing the level of histone acetylation and changes in chromatin configuration lead to restoring the expression of incorrectly silenced genes that are important for the correct functioning of the cell.

Studies on prostate cancer cells (BPH1, PC3, and LnCap) have demonstrated that sulforaphane acts as an inhibitor of histone deacetylase. Inhibition of HDAC activity was observed, with a simultaneous increase in histone acetylation in these cells. SFN supplementation resulted in slowing tumor growth as well as significant inhibition of HDAC in PC3 cells, in prostate cells, and in peripheral blood cells. Similar effects were observed for human colon cancer cells (HCT116) [50]. Other in vitro tests studied the influence of sulforaphane metabolites on HDAC activity. It turned out that the best inhibitive properties were demonstrated by the metabolites SFN-N-acetylcysteine and SFN-cysteine, which were obtained with molecular modeling techniques used in the active spot of the enzyme [51].

## 3. Materials and Methods

### 3.1. Equipment

Chromatograph HPLC 1100 (Agilent Technologies, Waldbronn, Germany) with C18 column ACE 5 C18-300 150 × 4.6 mm and pre-column ACE 5 C18-300 (VWR International, Radnor, PA, USA). Chromatograph LC-MS 8050, pump LC-30 AD, controller CBM 20A, autosampler SIL-30A, thermostat CTO-20AC (Shimadzu, Kyoto, Japan). Applied MS/MS conditions include electrospray ionization in positive (ESI+) and negative modes (ESI−), interface temperature of 300 °C, DL temperature of 250 °C, nebulizing gas flow of 3 L/min, heating gas flow of 10 L/min, and a drying gas temperature of 400 °C. Spectrophotometer UV-Vis Helios Gamma (Thermo Fisher Scientific, Waltham, MA, USA). Spectrophotometer—multifunctional reader Varioscan (ThermoScientific, Waltham, MA, USA). Inverted microscope with phase contrastym Olympus CKX41. SPE columns filled with silica gel (irregular shape), Superclean™ LC-Si SPE Tube bed wt. 100 mg, volume 1 mL (Merck, Darmstadt, Germany).

### 3.2. Chemicals and Reagents

Standards: sulforaphane ≥ 95% (HPLC), CAS 142825-10-3 (Sigma Aldrich, St. Louis, MO, USA). Reagents: 1,2-benzenedithiol 96%, 2,2-diphenyl-1-picrylhydrazyl (DPPH^•^) (Sigma Aldrich, St. Louis, MO, USA); disodium hydrogen phosphate, Na_2_HPO_4_ (ar grade), sodium dihydrogen phosphate, NaH_2_PO_4_ (ar grade), hydrochloric acid (ar grade) (Avantor S.A. Gliwice, Poland); medium for microbiological tests (DMEM), glutamine, bovine serum (FBS), and dimethyl sulfoxide (DMSO); MTT TOX1 set (Sigma-Aldrich, St. Louis, MO, USA). Organic solvents: methanol (ar grade), ethanol (ar grade) (Avantor S.A. Gliwice, Poland), and acetonitrile for HPLC from Sigma Aldrich (St. Louis, MO, USA).

### 3.3. Investigated Material

The plant material used in the presented research was broccoli (*Brassica oleracea* L.) at various stages of growth. Broccoli sprouting seeds (a certified eco-farming product) from Dary Natury, Grodzisk, Poland. Broccoli sprouts, trade name Vital Fresh, are produced by Jeronimo Martins S.A., Kostrzyn, Poland. Mature, shop-purchased broccoli heads are grown in Poland.

### 3.4. Preliminary Preparation of Plant Materials

Broccoli sprouts were grown from ecological seeds at room temperature. The water in the sprouting jar was changed several times a day—the seeds were washed with clean room-temperature water. After five days, the sprouts were harvested for further analysis.

### 3.5. Thermal Processing of Plant Samples

Sprouts grown in the lab, sprouts purchased in a shop, and a mature broccoli head were separately shredded, placed in string bags, and subjected to thermal processing—they were placed in a water bath heated to 60 °C for 5 min and 100 °C for 5 min.

The plant material, both raw and thermally processed, was subsequently frozen in liquid nitrogen while being ground in a mortar; the obtained material was then lyophilized. The procedures used are illustrated by Figure S11 (Supplementary File).

### 3.6. Isolation of Isothiocyanates from Plant Material—Preparation of Samples for HPLC/UV—Hydrolysis Reaction

Glucoraphanin was converted to sulforaphane via hydrolysis. To this end, 4 mL of acidified water (H_2_O + HCl 35–38%) with a strictly defined pH = 6 was added to ca. 150 mg of freeze-dried plant sample. The mixture was subsequently incubated at 45 °C for 2.5 h (Figure S12, Supplementary File).

After hydrolysis, the samples were extracted with 10 mL of dichloromethane, vortexed for several minutes, and stored for 1 h. After that time, the inorganic phase of the extract was separated from the organic one. Sulforaphane was purified with the use of disposable SPE columns packed with irregularly shaped silica gel Supelclean™ LC-Si SPE Tube bed wt. 100 mg, volume 1 mL (Merck, Darmstadt, Germany).

The columns were conditioned by gradually adding 3 mL of dichloromethane. Then 10 mL of the prepared plant extract was applied to the column, and the packing was washed with 3 mL of ethyl acetate, which was subsequently removed. Sulforaphane was eluted with 1 mL of methanol; the methanol extract was fully evaporated in a vacuum vortex evaporator and again dissolved in 1 mL of acetonitrile. The obtained solution was vortexed and then filtered with syringe filters.

### 3.7. Preparation of Samples for Determining Isothiocyanate Content

#### 3.7.1. Maceration with Methanol

Portions of ca. 150.0 mg of plant material were weighed and placed in plastic tubes (vol. 15 mL). The plant material was extracted with 3 mL of methanol for 24 h. To separate solid remains, the extract was then filtered through a paper filter on a rapid filtration funnel. The extracts were collected into plastic tubes and stored in a fridge.

#### 3.7.2. Maceration with Ethanol

Portions of ca. 200.0 mg of plant material were weighed and placed in plastic tubes. The plant material was extracted with 5 mL of ethanol for 24 h. To separate solid remains, the extract was then filtered through a paper filter on a rapid filtration funnel. The extracts were collected into plastic tubes and evaporated until dry.

#### 3.7.3. Extraction with Supercritical Fluid (SFE)

Portions of ca. 600.0 mg of plant material were weighed and placed in extraction cells together with filler material (glass beads). The plant material was subjected to extraction by supercritical carbon dioxide with an organic modifier (ethanol, 96%). Ca. 15 mL of ethanol extract was obtained in this way. The extraction conditions are presented in Table S2 (supplementary file).

#### 3.7.4. Extracts Prepared for the Analysis of Antioxidant Activity

To determine antioxidant activity, methanol solutions were prepared. Samples (150 mg each) were prepared from lyophilized materials 1 to 9 and from different sprouts (lab-grown broccoli sprouts as well as shop-purchased radish, broccoli, and kale sprouts), dried at 30 °C, and ground. The samples were macerated with 3 mL of methanol for 24 h at room temperature and then centrifuged; the supernatant was filtered through a syringe filter (PTFE, 13 mm diameter, 0.22 μm pore size).

### 3.8. Methods of Determining the Content of the Selected Isothiocyanate Sulforaphane in the Obtained Plant Extracts—Quantitative Analysis with HPLC/UV

The conditions of the analyses are presented in Table S3 (supplementary file). The analyses were carried out in isocratic conditions.

### 3.9. Method Validation

To validate the method, a calibration curve was constructed based on triple analyses of all seven dilutions with a concentration range of 4.0–80 µg/mL. The linearity of the curve was determined based on the coefficient of determination, r^2^. For the solution with the lowest concentration (4 µg/mL), the analysis was replicated seven times. Based on the calibration curve equation and the obtained chromatogram, the following parameters of the method were determined: precision, selectivity, repeatability, limit of detection (LOD), and limit of quantification (LOQ). The limit of detection describes the lowest concentration of the analyzed substance that can be detected when using a specific analytical technique with a specific probability. The LOD in this study was calculated based on the constructed calibration curve according to the equation below:

$$LOD=\frac{3.3\cdot SD}{a}$$

where

a—the slope of calibration curve
SD—standard deviation

Limit of quantification is the smallest amount or the lowest concentration of a substance that can be quantified with the specific analytical method with the assumed accuracy and precision. The LOQ was calculated with the following equation:

$$LOQ=\frac{10\cdot SD}{a}$$

### 3.10. Sample Analysis

In order to determine sulforaphane in the prepared extracts, 10 µL of an investigated sample was injected into a column. Each extract was analyzed in duplicate. Based on the model retention time, the sulforaphane peak was identified in the investigated plant samples. Peak purity was also confirmed by characteristic chromatograms obtained for the pure standard solution and for the tested samples. Quantitative analysis used the external standard method, which focuses on determining the relationship between the peak area and the concentration of a determined substance. To determine the sulforaphane content in the investigated extracts, the peak areas were correlated with the concentrations determined for the calibration curve. The sulforaphane content in samples was described as the average ± standard deviation.

### 3.11. LC/MS Qualitative Analysis Confirming the Presence of Sulforaphane and Its Metabolites in the Tested Plant Extracts

MS/MS analyses were carried out with the use of electrospray ionization in positive (ESI+) and negative (ESI−) modes. Analyses in tandem with LC were conducted with chromatographic column ACE 5 C18-300 (150 × 4.6 mm), mobile phase: acetonitrile/water (30:70 *v*/*v*), with formic acid added to water (0.1% HCOOH). Other working parameters of the mass spectrometer are interface temperature of 300 °C, DL temperature of 250 °C, nebulizing gas flow 3 L/min, heating gas flow of 10 L/min, and drying gas temperature of 400 °C.

### 3.12. Determining General Isothiocyanate Content by Cyclocondensation with 1,2-Benzenedithiol

This method uses the property of isothiocyanates to react quantitatively with 1,2-benzenedithiol. This reaction produces cyclic thiocarbonyl and free amines (Figure S13, supplementary file). The mechanism of the cyclocondensation reaction is based on the strongly electrophilic property of carbon in the isothiocyanate –NCS group, which is attacked first by one and then by the other thiol group in the dithiol structure [32].

Quantitative determination of isothiocyanates via cyclocondensation is based on the ability of the created 1,3-benzenedithiole-2-thione to absorb waves in the UV range. The maximum absorbance was determined for the wavelength of 365 nm [31].

**Figure 8:**
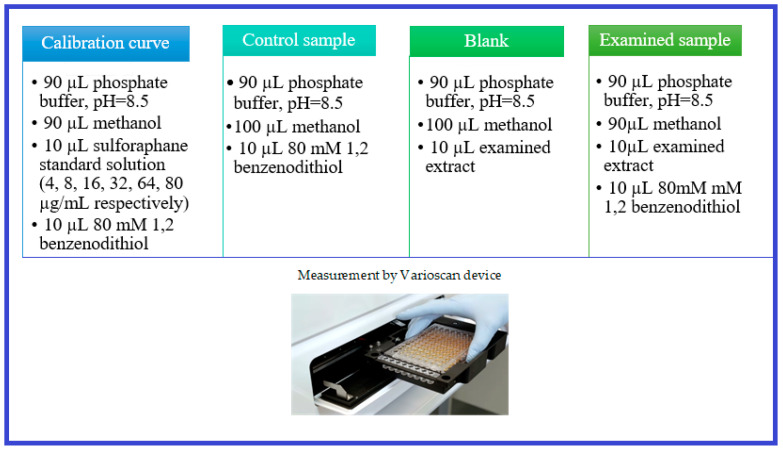
Preparation of samples for the analysis of isothiocyanates via cyclocondensation with 1,2-benzenedithiol.

In order to determine isothiocyanates in the analyzed extracts, a standard solution of 1,2-benzenedithiol at a concentration of 80 mM was prepared. To this end, 114.2 mg of the standard reagent was dissolved in 10 mL of methanol. The analysis was carried out on a 96-well plate. The reaction mixture with the ultimate volume of 200 µL was prepared in the following way (Figure 8):

The solutions were applied to a 96-well plate with a pipette according to the system presented above, which allowed for a duplicate test of each extract. In order to initiate the reaction, the last reagent added was 1,2-benzenedithiol. Then the plate was heated without light access at 65 °C for 60 min. After that hour, the plate was left to cool and then placed in a Varioscan device, where the absorbance values were read at the wavelength λ = 365 nm.

### 3.13. Assessment of the Antioxidant Activity of Studied Plant Extracts with DPPH^•^ Used as Reagent

The antioxidant activity of specific extracts was determined by the commonly used DPPH^•^ method of capturing free radicals. For this purpose, 100 µL of each extract was poured into 5 mL vials made of dark glass, and 3.0 mL of DPPH^•^ reagent (methanol solution 0.02 mg/mL) was added to each vial. The samples were left in the darkness for 30 min. Then, for the wavelength λ = 517 nm, absorbance was determined for individual mixtures and for pure DPPH^•^ solution. For a blind test, pure methanol was used. The methanol solution of the DPPH^•^ reagent is purple, and due to the reduction of the radical to DPPH-H, the solution changed color to yellow-green. The measured absorbance values were used to calculate the antioxidant activity (RSA) according to the following formula:

$$RSA=\frac{A_{DPPH}-A}{A_{DPPH}}\cdot 100\%$$

where RSA—%RSA (radical scavenging activity)—ability to sweep free radicals; A—the absorbance of tested solution; A_DPPH_—absorbance of DPPH^•^.

The obtained results were subjected to statistical analysis. The above method of determining antioxidant activity is based on the mechanism of single electron transfer (SET), which, generally speaking, involves introducing both an antioxidant and an agent undergoing reduction into the reaction environment. The transfer of an electron from antioxidant to oxidant leads to a change in the color of the reaction mixture. When the color stops changing, the reaction has reached its endpoint. The difference in absorbance as a function of antioxidant concentration in the tested solution is usually linear, so the results obtained for real samples can be calculated as Trolox equivalent (TE). Trolox (6-hydroxy-2,5,7,8-tetramethylchroman-2-carboxylic acid) is a standard substance that has high antioxidant properties and is soluble in water. To calculate TEAC (Trolox Equivalent Antioxidant Capacity), the authors used data obtained for standard solutions: Trolox concentration ranges from 0.1 to 0.8 mM; calibration curve equation y = −0.4655x + 0.5012; coefficient of determination R^2^ = 0.9942.

### 3.14. Assessment of Cytotoxic Activity of Extracts Obtained from Selected Cruciferous Plants, Conducted by Use of the MTT Assay

To determine the cytotoxic activity of the tested extracts, the MTT colorimetric assay was used. For the analysis of cytotoxic potential, the authors selected the cell lines of mouse fibroblast L929 (European Collection of Authenticated Cell Cultures) and human colon adenocarcinoma Caco-2. The cells were cultured in DMEM with 2 mM of glutamine and 10% fetal bovine serum (FBS) with the addition of 1% (*v*/*v*) of penicillin and streptomycin solution (10,000 units of penicillin and 10 mg of streptomycin/mL) at 37 °C in a humidified incubator in the atmosphere of 5% CO_2_ in order to obtain a merged layer of cells. The cells were separated from the medium with 0.25% trypsin and 100 µL of cell suspension with a density of 2 × 10^5^ cells/mL, and then transferred to a 96-well plate and incubated for 24 h at 37 °C in 5% CO_2_.

From the obtained plant extracts, starting solutions with a concentration of 50 mg/mL were prepared by dissolving the dry residue in DMSO. The starting solutions were used to make a series of dilutions in culture medium so as to obtain a series of concentrations of 0.5–5000 µg/mL. Next, the medium in the 96-well plate containing L929 and Caco-2 cells was replaced with the medium containing the tested extracts in the abovementioned range of concentrations, and the plate was incubated for 48 h. Cells in fresh culture medium without extract were considered control samples.

After 48 h, 10 µL of the MTT TOX1 set was added to each well and stirred. After 3.5 h of incubation at 37 °C, the medium was carefully removed, and isopropanol solution was added to dissolve the formazan crystals that had emerged. Absorbance was read for following wavelengths: 570 and 620 nm for background with a microplate reader (Multiskan, ThermoFisher). All tests were made in triplicate, and the relative cell viability (IC50) was calculated. The MTT cytotoxicity test was performed for the following samples: E1—raw broccoli sprouts; E3—raw broccoli head; E4—broccoli sprouts processed at 60 °C; E6—broccoli head processed at 60 °C; E7—broccoli sprouts processed at 100 °C; and E9—broccoli head processed at 100 °C.

## 4. Conclusions

The high content of isothiocyanates, including sulforaphane, in both sprouts and mature broccoli heads has been confirmed. In addition to anti-cancer activity, sulforaphane was also studied with regard to its other biological activities. Sulforaphane is able to activate some factors that play a pivotal role in protecting cells against oxidative damage, generating such conditions as tumors, diabetes, cardiovascular diseases, neurodegenerative diseases, and chronic diseases of the kidneys and liver [18]. Benefits from administering sulforaphane in neurodegenerative diseases are related to the transcription factor Nrf2 signaling pathway, whose activator is SFN. The available data confirms that the anti-oxidative activity of SFN resulting from this mechanism protects immature neurons against death caused by ischemic and traumatic brain damage [52]. The ability to reduce oxidative stress and anti-inflammatory activity inspired research on the impact of daily doses of sulforaphane on the behavior of patients with autism spectrum disorder. In the group of people receiving a daily dose of sulforaphane, an improvement was observed in social interactions, correct behavior, and verbal communication [53]. All these complex mechanisms, not only chemopreventive and anti-cancer ones but also anti-inflammatory and protecting against oxidative damage, make sulforaphane a promising compound that demonstrates numerous health benefits [41,54]. Hence, analytical and clinical research should be continued with a view to developing phytopharmaceuticals containing highly available sulforaphane.

## Figures and Tables

**Table 1:** Summary of the *m/z* ratio of pseudo-molecular ions and product ions for individual compounds determined with the LC/MS technique.

	Compound	General Formula	Ionization Mode	Pseudomolecular Ion *m/z*	Daughter Ions *m/z*
	Sulforaphane (SFN)	C_6_H_11_NOS_2_	ESI+	178.25	55.05 72.10 114.05 119.15
**Metabolites**	Sulforaphane-cysteine (SFN-CYS)	C_9_H_18_N_2_O_3_S_3_	ESI+	299.05	109.20 130.10 150.20 165.10 189.30 211.05 238.05 252.75 269.80 280.95
	Sulforaphane- glutathione (SFN-GSH)	C_16_H_28_N_4_O_7_S_3_	ESI+	485.10	155.20 215.20 243.10 268.00
	Sulforaphane-N- acetyl-cysteine (SFN-NAC)	C_11_H_2_0N_2_O_4_S_3_	ESI+	341.65	147.00 192.00 217.30 238.70 255.20 279.20 299.95 322.90
	Sulforaphane- cysteinoglycin (SFN-CG)	C_11_H_21_N_3_O_4_S_3_	ESI+	356.20	114.90 194.20 232.80 272.90 285.10 303.40 314.40 338.00
	Glucoraphanin (GRN)	C_12_H_23_NO_10_S_3_	ESI−	436.00	176.40 288.20 398.90

Based on the *m/z* signals from product ions, the authors proposed potential fragmentation paths for sulforaphane (supplementary file, Figure S4), its metabolites (supplementary file, Figures S5–S8), and glucoraphanin (supplementary file, Figure S9).

**Table 2:** Comparison of signal intensity in mass spectra obtained for the tested samples. SIM mode.

Signal Intensity, SIM Mode						
Type of Sample	Sulforaphane (SFN)	Sulforaphane -Cysteine (SFN-CYS)	Sulforaphane -Glutathione (SFN-GSH)	Sulforaphane- N-acetyl-cysteine (SFN-NAC)	Sulforaphane- cysteinoglycin (SFN-CG)	Glucoraphanin (GRN)
	178 *m/z*	299 *m/z*	485 *m/z*	341 *m/z*	356 *m/z*	436 *m/z*
E1, sprouts grown in the lab	7.5 × 10^4^	3.0 × 10^4^	3.0 × 10^4^	1.0 × 10^5^	3.0 × 10^4^	2.0 × 10^4^
E2, sprouts purchased in a shop	3.0 × 10^5^	2.5 × 10^4^	1.5 × 10^4^	3.0 × 10^4^	3.0 × 10^4^	n/o
E3, mature broccoli head	2.0 × 10^4^	7.5 × 10^4^	2.5 × 10^4^	7.0 × 10^4^	4.0 × 10^4^	1.5 × 10^3^
E4, sprouts grown in the lab, treated temp. 60 °C	3.0 × 10^4^	7.0 × 10^4^	3.0 × 10^4^	6.0 × 10^4^	5.0 × 10^4^	2.5 × 10^3^
E5, sprouts purchased in a shop, treated temp. 60 °C	9.5 × 10^4^	2.5 × 10^4^	9.0 × 10^3^	3.5 × 10^4^	3.0 × 10^4^	1.0 × 10^3^
E6, mature broccoli head, treated temp. 60 °C	3.0 × 10^5^	8.0 × 10^4^	2.5 × 10^4^	5.0 × 10^4^	3.5 × 10^4^	2.0 × 10^3^
E7, sprouts grown in the lab, treated temp. 100 °C	2.5 × 10^6^	6.0 × 10^4^	2.0 × 10^4^	5.0 × 10^4^	5.0 × 10^4^	1.5 × 10^3^
E8, sprouts purchased in a shop, treated temp. 100 °C	1.0 × 10^6^	4.0 × 10^4^	1.5 × 10^4^	3.0 × 10^4^	3.5 × 10^4^	2.0 × 10^3^
E9, mature broccoli head, treated temp. 100 °C	7.5 × 10^4^	2.0 × 10^4^	1.5 × 10^4^	2.5 × 10^4^	2.0 × 10^4^	2.0 × 10^4^

n/o—not observed.

**Table 3:** Intensity values for MS signals obtained in SIM mode for sulforaphane and glucoraphanin in specific extracts.

Extract	SFN	GRN
	MS signals intensity based on SIM	
E1	75,000	20,000
E2	300,000	0
E3	20,000	1500
E4	30,000	2500
E5	95,000	1000
E6	300,000	2000
E7	2,500,000	1500
E8	1,000,000	2000
E9	75,000	20,000

**Table 4:** Validation parameters.

Parameter	Results
Specificity	detection: λ = 202 nm column: octadecyl ACE 5 C18-300 150 × 4.6 mm; pre-column ACE 5 C18-300 (VWR International, Radnor, PA, USA)
Linearity range [μg/mL]	4.00–80.00
Regression curve (y = ax + b)	y = 13.205x + 34.457
R^2^ coefficient of determination coefficient of the calibration curve for the standards used in the concentration range mentioned above	0.9989
SD (s) standard deviation of chromatographic peak areas of standard solution (the lowest concentration 4.00 μg/mL)	4.48
Precision: Relativee standard deviation, RSD	0.057
Repeatability: coefficient of variation, CV [%]	5.7
Limit of detection LOD [μg/mL] $\left(LOD=3.3\frac{s}{a}\right)$	1.12
Limit of quantification LOQ [μg/mL] $\left(LOQ=10.0\frac{s}{a}\right)$	3.39

**Table 5:** Summary of sulforaphane determination results for the tested plant extracts with HPLC/UV-Vis.

Extract	Average Sulforaphane Concentration [µg/g]	±SD
E1	6.46	3.18
E2	120.83	0.43
E3	≤LOQ	-
E4	≤LOQ	-
E5	≤LOQ	-
E6	57.26	0.43
E7	855	7
E8	352	5
E9	12.59	3.67

**Table 6:** Summary results obtained for tested samples of sprouts and mature broccoli exposed to various factors.

Extract	Average Sulforaphane Concentration c ± SD [mg/g]	Total Isothiocyanins Concentration c ± SD [mg/g]	RSA ± SD [%]	TEAC ± SD [mM]
E1	0.006 ± 0.001	2.278 ± 0.838	53.33 ± 0.14	0.350 ± 0.002
E2	0.121 ± 0.004	1.580 ± 0.256	51.81 ± 0.14	0.330 ± 0.002
E3	≤LOQ	0.542 ± 0.178	13.98 ± 0.08	0.092 ± 0.001
E4	≤LOQ	2.685 ± 0.237	54.07 ± 0.16	0.370 ± 0.002
E5	≤LOQ	2.428 ± 0.055	58.10 ± 0.08	0.430 ± 0.001
E6	0.057 ± 0.001	1.042 ± 0.204	14.26 ± 0.08	0.095 ± 0.001
E7	0.855 ± 0.007	2.083 ± 0.096	75.28 ± 0.10	0.690 ± 0.004
E8	0.352 ± 0.005	1.046 ± 0.153	53.43 ± 0.08	0.360 ± 0.001
E9	0.013 ± 0.001	0.517 ± 0.046	37.50 ± 0.12	0.110 ± 0.002

**Table 7:** Cytotoxic activity of extracts obtained from cruciferous plants towards the cell line of mouse fibroblasts L929 and colon adenocarcinoma cells Caco-2.

	**% Viability ± SD** **Cell Line L929**				
	**Concentration [µg/mL]**				
**Extract**	**0.5**	**5**	**50**	**500**	**5000**
E1 raw sprouts grown in the lab	134 ±10	141 ±4	121 ±14	128 ±6	55 ±3
E4 sprouts at 60 °C	147 ±11	137 ±7	151 ±2	130 ±5	36 ±2
E7 sprouts at 100 °C	131 ±6	133 ±9	127 ±12	114 ±7	87 ±3
E3 raw mature broccoli head	118 ±18	140 ±3	115 ±4	112 ±17	50 ±2
E6 broccoli at 60 °C	157 ±12	166 ±4	132 ±21	150 ±13	59 ±5
E9 broccoli at 100 °C	135 ±3	130 ±5	122 ±8	123 ±3	57 ±4
	**% Viability ± SD** **Cell Line Caco-2**				
	**Concentration [µg/mL]**				
**Extract**	**0.5**	**5**	**50**	**500**	**5000**
E1 raw sprouts grown in the lab	100 ±3	138 ±2	117 ±8	118 ±5	60 ±6
E4 sprouts at 60 °C	109 ±6	102 ±19	110 ±5	114 ±9	74 ±1
E7 sprouts at 100 °C	81 ±22	102 ±7	119 ±7	115 ±7	99 ±1
E3 raw mature broccoli head	108 ±11	134 ±19	132 ±7	122 ±4	45 ±6
E6 broccoli at 60 °C	113 ±8	110 ±8	116 ±2	97 ±5	58 ±1
E9 broccoli at 100 °C	144 ±4	107 ±2	78 ±0.1	90 ±6	57 ±4

## Data Availability

Data are contained within the article or Supplementary Material.

## Supplementary Materials

The following supporting information can be downloaded at: www.mdpi.com/xxx/s1, Figure S1. Sulforaphane calibration curve; Figure S2. Summarize the fragmentation spectra obtained for the standard solution of sulforaphane; Figure S3. Summarize the fragmentation spectra obtained for the standard solution of sulforaphane’s metabolites; Figure S4. Proposed potential fragmentation paths of sulforaphane; Figure S5. Proposed potential fragmentation paths of sulforaphane’s metabolite: sulforaphane-cysteine; Figure S6. Proposed potential fragmentation paths of sulforaphane’s metabolite: sulforaphane-glutathione; Figure S7. Proposed potential fragmentation paths of sulforaphane’s metabolite: sulforaphane-N-acetyl-cysteine; Figure S8. Proposed potential fragmentation paths of sulforaphane’s metabolite: sulforaphane-cysteinoglycin; Figure S9. Proposed potential fragmentation paths of sulforaphane’s metabolite: glucoraphanin; Figure S10. Example mass spectra with characteristic signals, obtained for the extract of raw lab-grown broccoli sprouts (E1). Figure S11. Diagram of sample preparation steps. Figure S12. Hydrolysis of glucoraphanin. Figure S13. Cyclocondensation of isothiocyanates with 1,2-benzenedithiol. Table S1. The summary of absorbance values measured for individual samples as well as the calculated isothiocyanate concentrations (µg/mL); Table S2. Conditions of supercritical fluid extraction (SFE). Table S3. Conditions of HPLC/UV analysis.
