# Supplementary material for: Comparative Studies of Extracts Obtained from Brassica oleracea L. Plants at Different Stages of Growth by Isolation and Determination of Isothiocyanates: An Assessment of Chemopreventive Properties of Broccoli
Source: Molecules. 2024 Jan 20;29(2):0. doi: 10.3390/molecules29020519 (PMC11154519; doi:10.3390/molecules29020519)
Supplement: Supplementary file 1 [file molecules-29-00519-s001.zip › molecules-2809633-supplementary.pdf]

Supplementary Materials

The comparative studies of extracts obtained from Brassica oleracea L. plants in different stages of growth by the isolation and determination of isothiocyanates – an assessment of chemopreventive properties

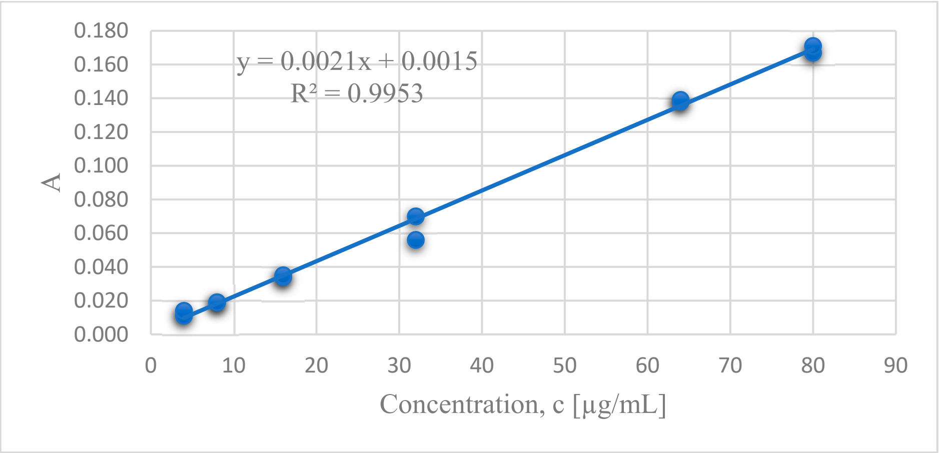

Figure S1. Sulforaphane calibration curve.

Table S1. The summary of absorbance values measured for individual samples as well as the calculated isothiocyanate concentrations (μg/mL).

	Sample	A1	A2	C1	C2	C_{av} [μg/mL]	± SD
Methanolic extracts	E1, sprouts grown in the lab, lyophilizate	0,309	0,182	146,43	85,95	116,19	42,76
	E2, sprouts purchased in a shop, lyophilizate	0,152	0,191	71,67	90,24	80,95	13,13
	E3, mature broccoli head, lyophilizate	0,046	0,073	21,19	34,05	27,62	9,09
	E4, sprouts grown in the lab at temp. 60 ^{0}C	0,308	0,272	145,95	128,81	137,38	12,12
	E5, sprouts purchased in a shop at temp. 60 ^{0}C	0,249	0,257	117,86	121,67	119,76	2,69
	E6, mature broccoli head at temp. 60 ^{0}C	0,125	0,095	58,81	44,52	51,67	10,10
	E7, sprouts grown in the lab at temp. 100 ^{0}C	0,210	0,224	99,29	105,95	102,62	4,71
	E8, sprouts purchased in a shop at temp. 100 ^{0}C	0,101	0,124	47,38	58,33	52,86	7,74
	E9, mature broccoli head at temp. 100 ^{0}C	0,054	0,061	25,00	28,33	26,67	2,36
SFE extracts	E1, sprouts grown in the lab, lyophilizate	0,607	0,505	288,33	239,76	264,05	34,35
	E3, mature broccoli head, lyophilizate	0,067	0,091	31,19	42,62	36,90	8,08
	E6, mature broccoli head at temp. 60 ^{0}C	0,238	0,281	112,62	133,10	122,86	14,48
Ethanolic extracts	E1, sprouts grown in the lab, lyophilizate	0,064	0,081	29,76	37,86	33,81	5,72
	E3, mature broccoli head, lyophilizate	0,052	0,058	24,05	26,90	25,48	2,02
	E6, mature broccoli head at temp. 60 ^{0}C	0,014	0,011	5,95	4,52	5,24	1,01

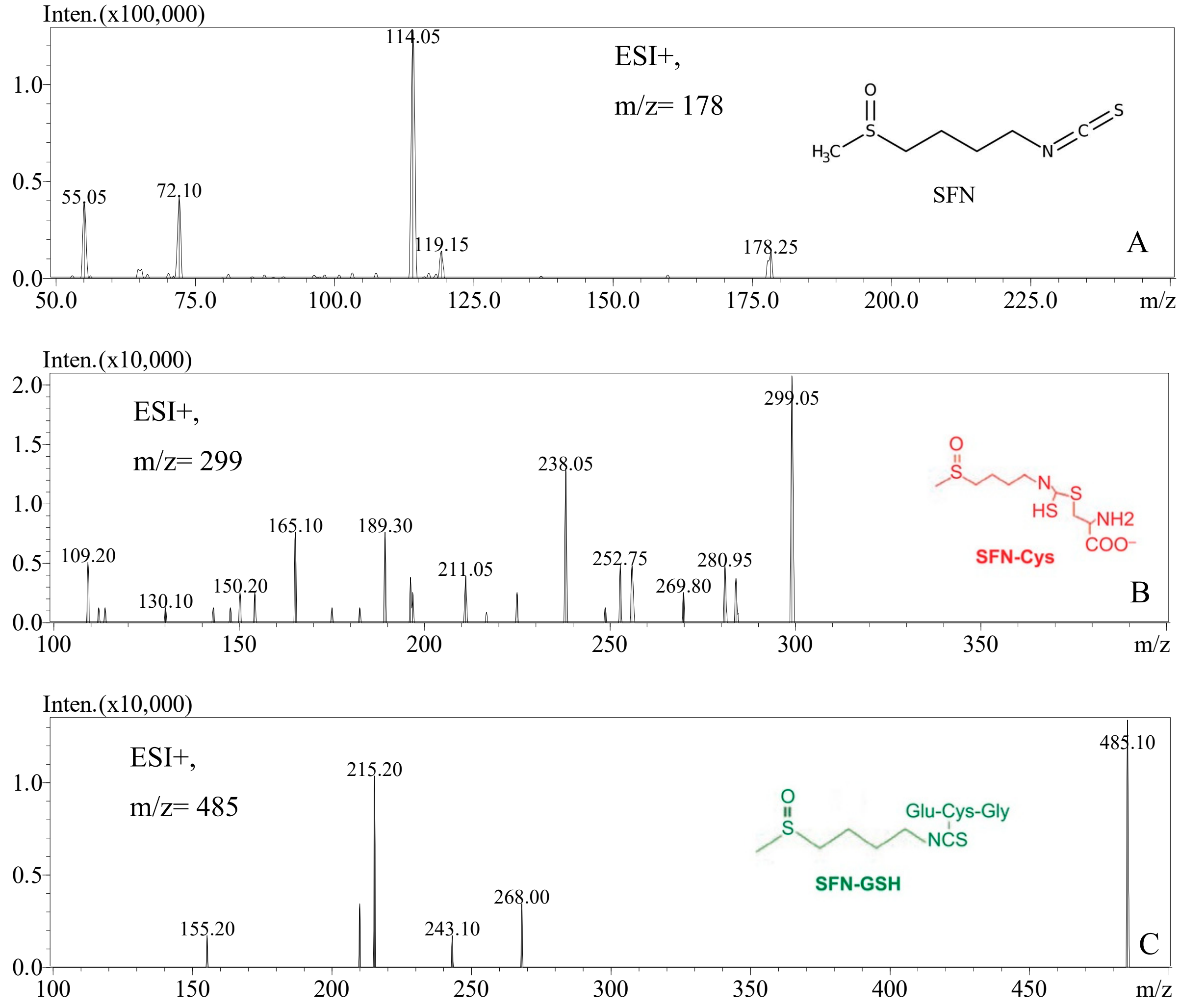

Figure S2. Summarize the fragmentation spectra obtained for the standard solution of sulforaphane.

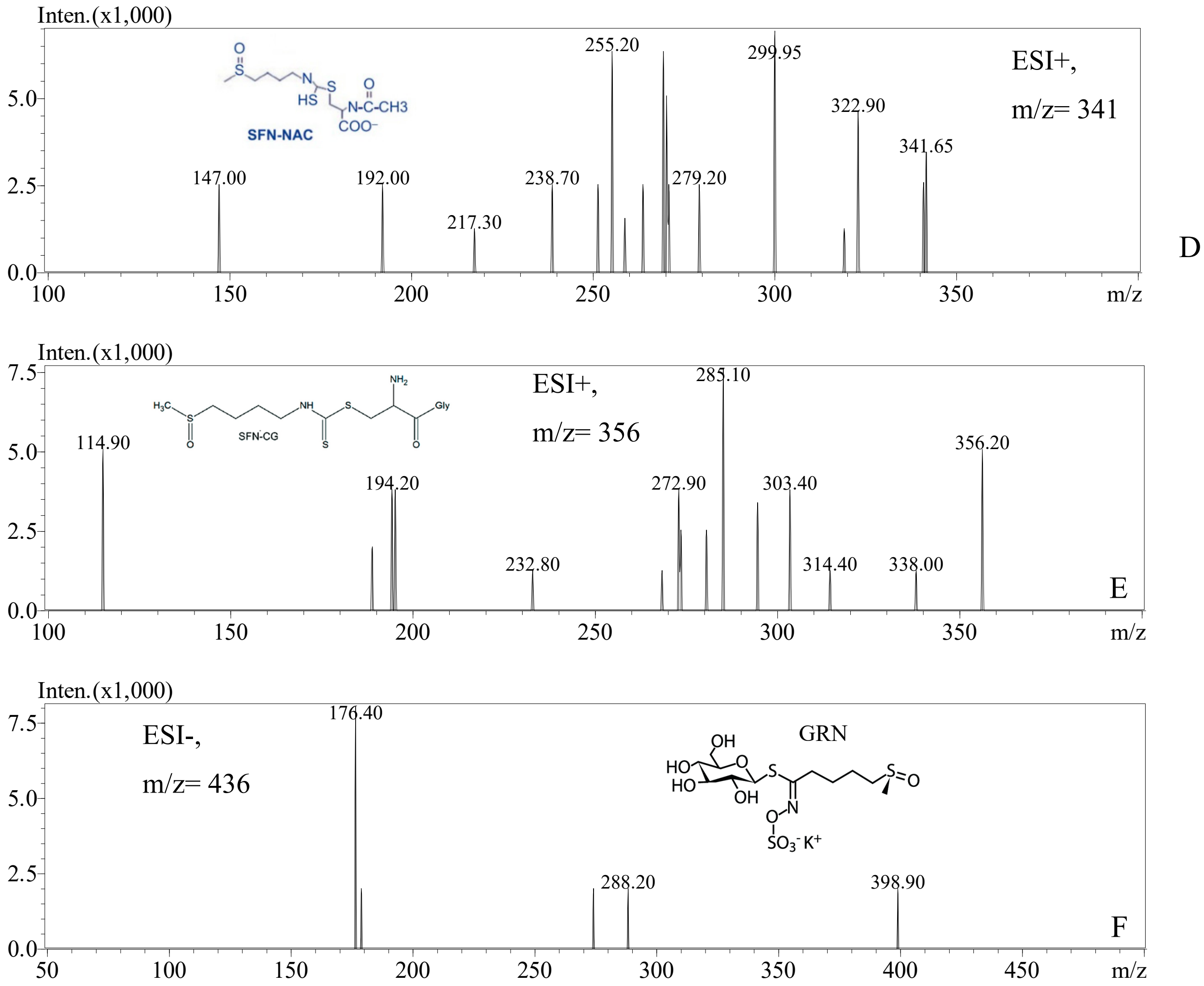

Figure S3. Summarize the fragmentation spectra obtained for the standard solution of sulforaphane's metabolites.

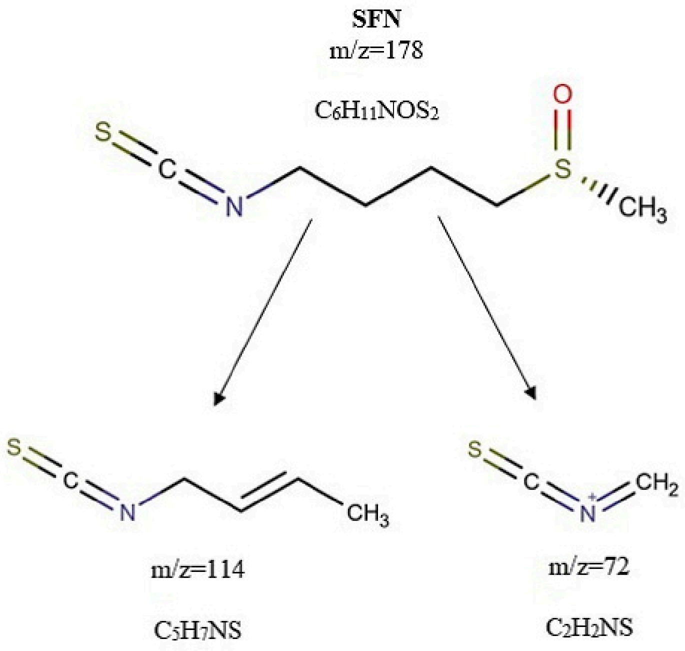

Figure S4. Proposed potential fragmentation paths of sulforaphane.

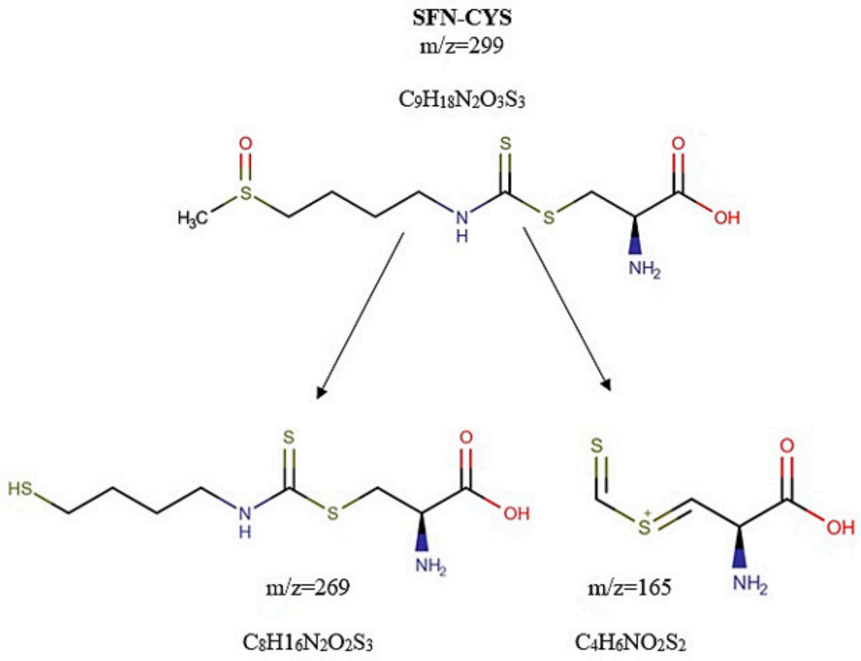

Figure S5. Proposed potential fragmentation paths of sulforaphane's metabolite: sulforaphane-cysteine.

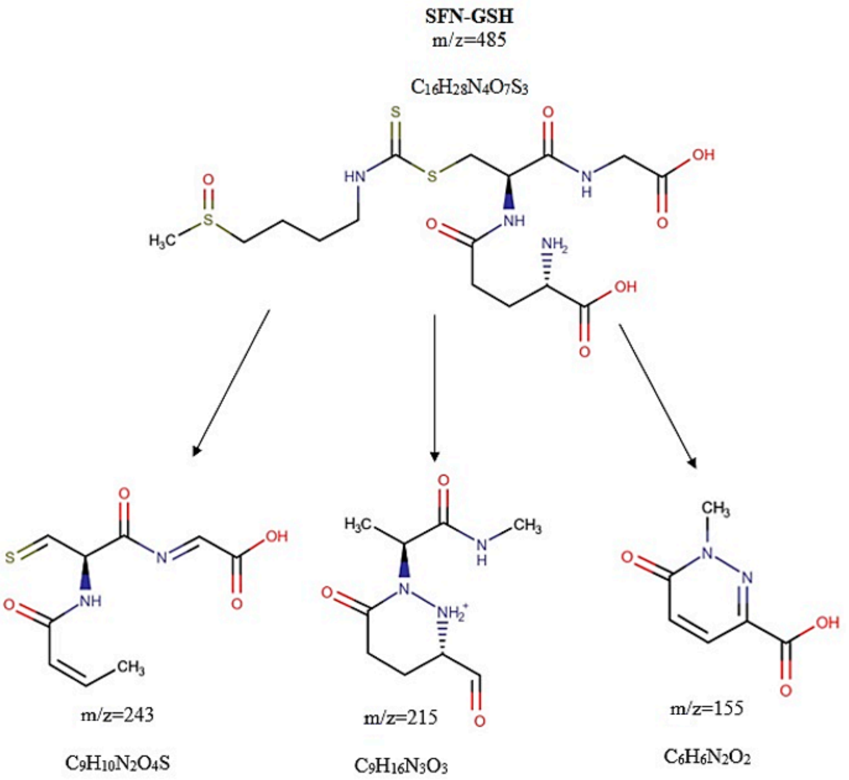

Figure S6. Proposed potential fragmentation paths of sulforaphane's metabolite: sulforaphane-glutathione.

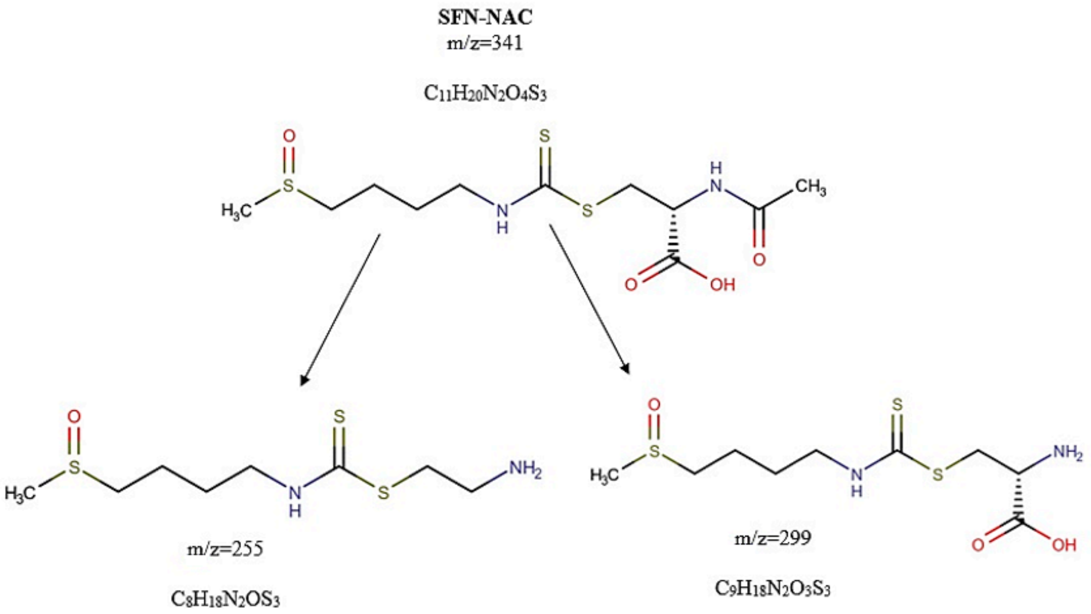

Figure S7. Proposed potential fragmentation paths of sulforaphane's metabolite: sulforaphane-N-acetyl-cysteine.

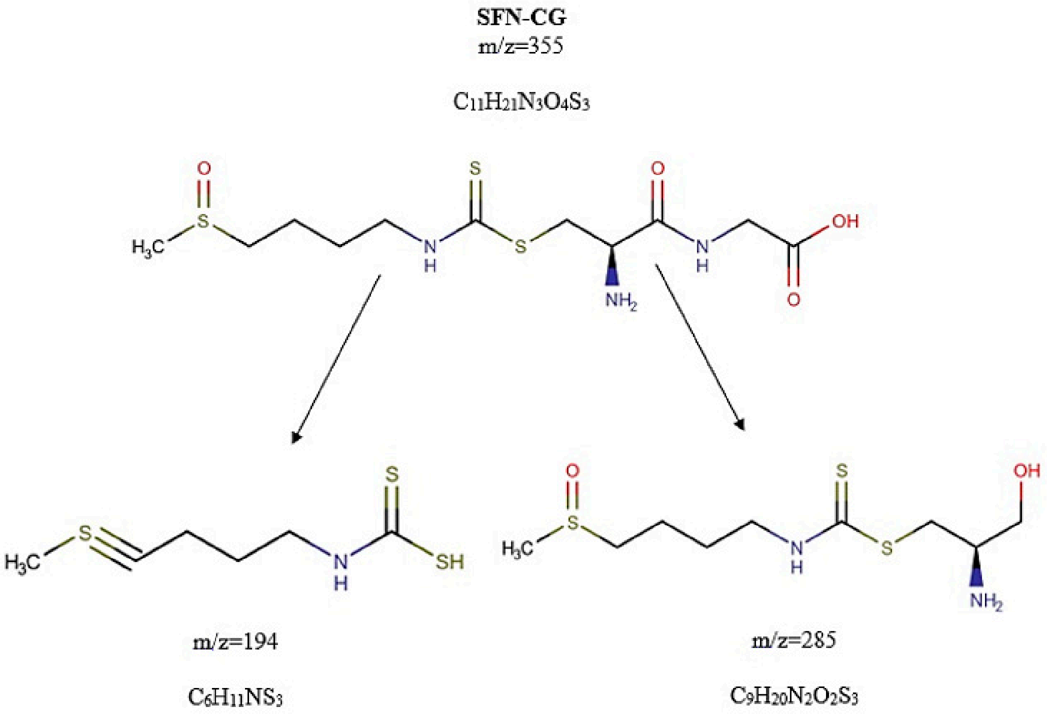

Figure 8S. Proposed potential fragmentation paths of sulforaphane's metabolite: sulforaphane-cysteinoglycin

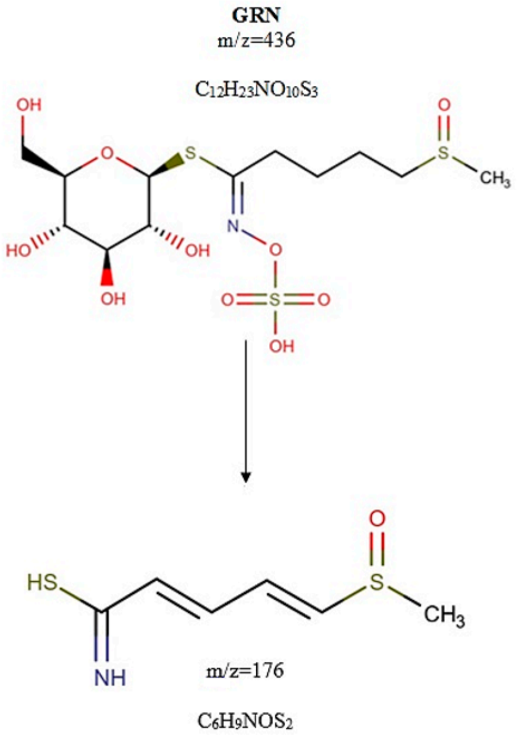

Figure S9. Proposed potential fragmentation paths of sulforaphane's metabolite: glucoraphanin.

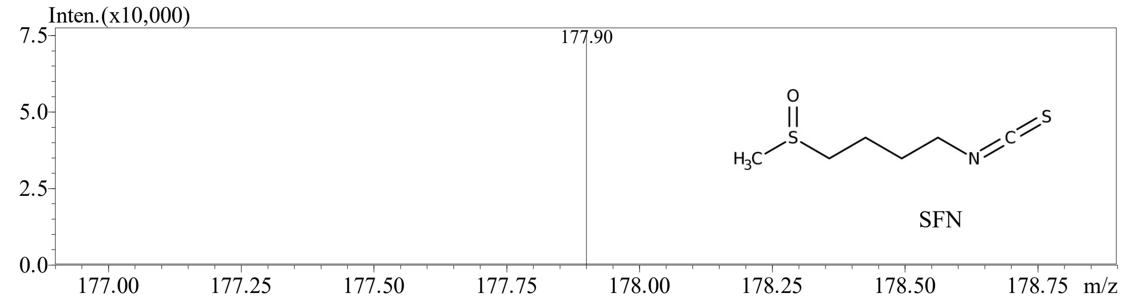
Inten.(x10,000)
7.5
5.0
2.5
0.0
177.90
177.00
177.25
177.50
177.75
178.00
178.25
178.50
178.75
m/z
SFN

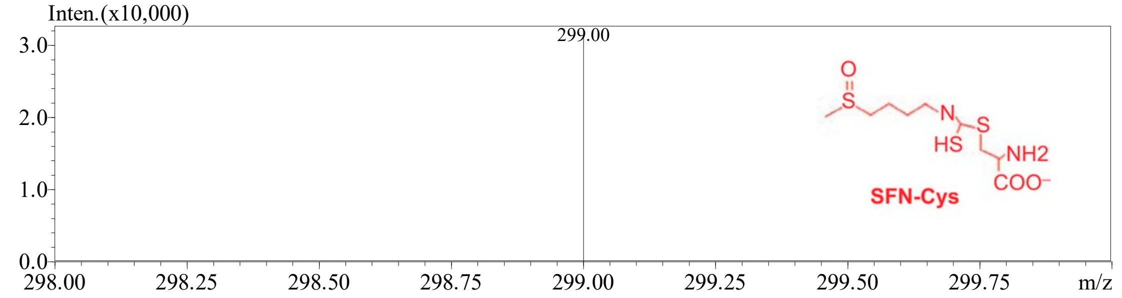
Inten.(x10,000)
3.0
2.0
1.0
0.0
299.00
298.00
298.25
298.50
298.75
299.00
299.25
299.50
299.75
m/z
SFN-Cys

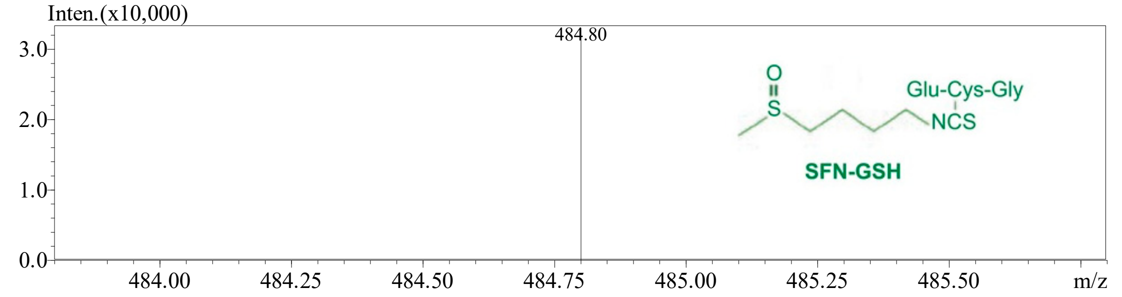
Inten.(x10,000)
3.0
2.0
1.0
0.0
484.80
484.00
484.25
484.50
484.75
485.00
485.25
485.50
m/z
Glu-Cys-Gly
SFN-GSH

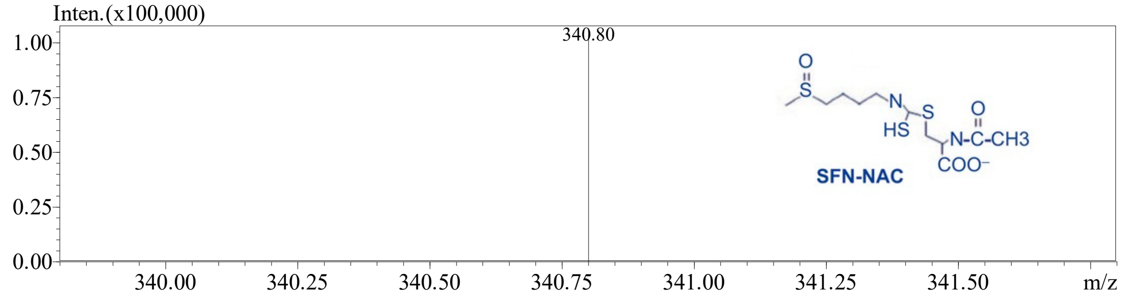
Inten.(x100,000)
1.00
0.75
0.50
0.25
0.00
340.80
340.00
340.25
340.50
340.75
341.00
341.25
341.50
m/z
SFN-NAC

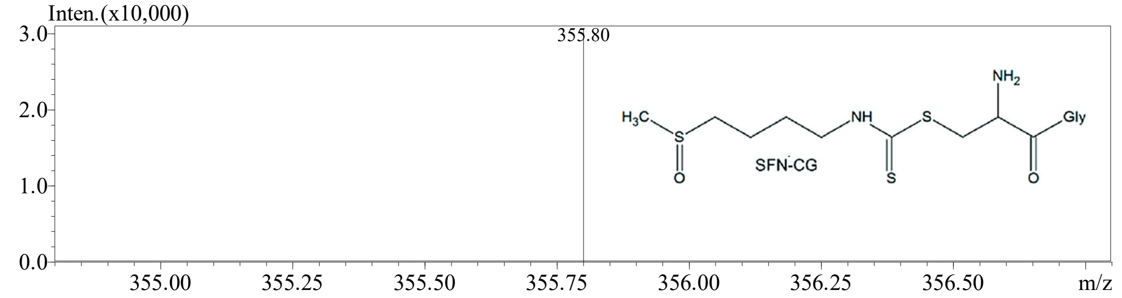
Inten.(x10,000)
3.0
2.0
1.0
0.0
355.80
355.00
355.25
355.50
355.75
356.00
356.25
356.50
m/z
SFN-CG

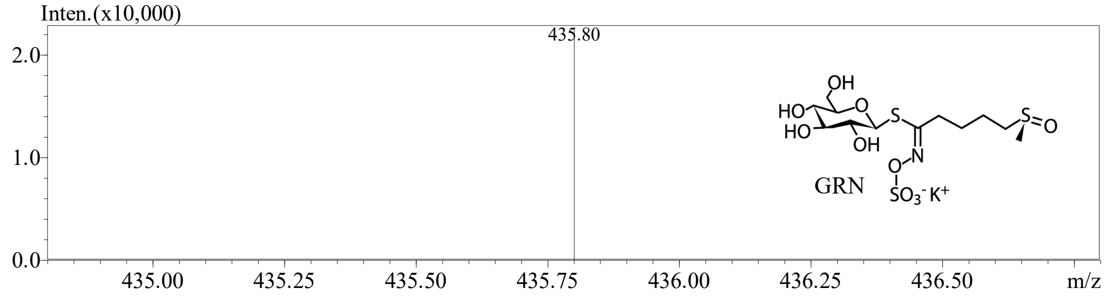

Figure S10. Example mass spectra with characteristic signals, obtained for the extract of raw lab-grown broccoli sprouts (E1).

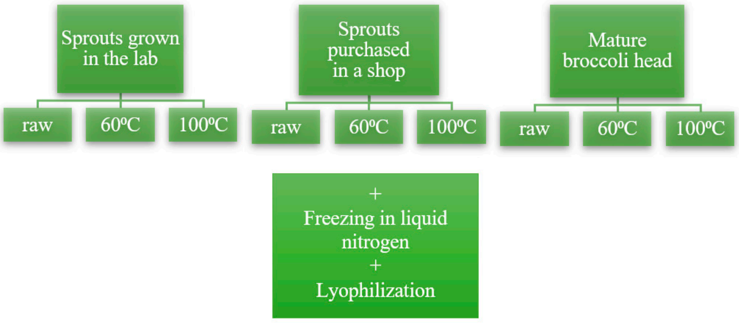

Figure S11. Diagram of sample preparation steps.

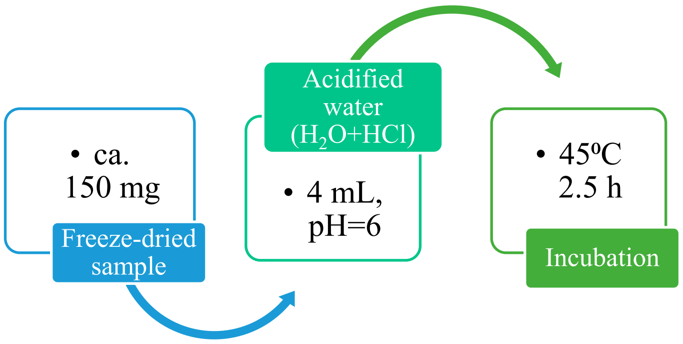

Figure S12. Hydrolysis of glucoraphanin.

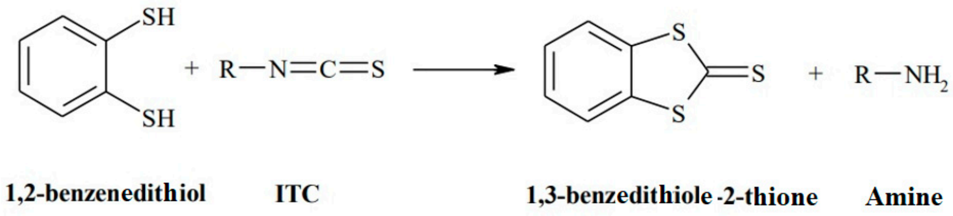

Figure S13. Cyclocondensation of isothiocyanates with 1,2-benzenedithiol.

Table S2. Conditions of supercritical fluid extraction (SFE).

Pressure	200 bar
Temperature	50°C
CO_2 flow	4.0 mL/min
Co-solvent flow rate (ethanol, 96%)	1.0 mL/min
Extraction time	60 min (static)<br>10 min (dynamic)

Table S3. Conditions of HPLC/UV analysis.

Type of chromatography column	Octadecyl ACE 5 C18-300,<br>pre-column ACE 5 C18-300<br>(VWR International, Radnor, PA, USA)
Column dimensions	150 × 4.6 mm
Mobile phase	Acetonitrile/water (30:70, *v*/*v*)
Flow rate	0.6 mL/min
Injection volume	10 µL
UV detector wavelength	202 nm
